# Elementary methods provide more replicable results in microbial differential abundance analysis

**DOI:** 10.1093/bib/bbaf130

**Published:** 2025-03-26

**Authors:** Juho Pelto, Kari Auranen, Janne V Kujala, Leo Lahti

**Affiliations:** Department of Computing, University of Turku, University of Turku, 20014, Finland; Department of Mathematics and Statistics, University of Turku, University of Turku, 20014, Finland; Department of Mathematics and Statistics, University of Turku, University of Turku, 20014, Finland; Department of Clinical Medicine, University of Turku, University of Turku, 20014, Finland; Department of Mathematics and Statistics, University of Turku, University of Turku, 20014, Finland; Department of Computing, University of Turku, University of Turku, 20014, Finland

**Keywords:** differential abundance analysis, microbiome, benchmarking, replicability

## Abstract

Differential abundance analysis (DAA) is a key component of microbiome studies. Although dozens of methods exist, there is currently no consensus on the preferred methods. While the correctness of results in DAA is an ambiguous concept and cannot be fully evaluated without setting the ground truth and employing simulated data, we argue that a well-performing method should be effective in producing highly reproducible results. We compared the performance of 14 DAA methods by employing datasets from 53 taxonomic profiling studies based on 16S rRNA gene or shotgun metagenomic sequencing. For each method, we examined how the results replicated between random partitions of each dataset and between datasets from separate studies. While certain methods showed good consistency, some widely used methods were observed to produce a substantial number of conflicting findings. Overall, when considering consistency together with sensitivity, the best performance was attained by analyzing relative abundances with a nonparametric method (Wilcoxon test or ordinal regression model) or linear regression/*t*-test. Moreover, a comparable performance was obtained by analyzing presence/absence of taxa with logistic regression.

## Introduction

Studying associations between microbial taxa and external variables, such as disease status or environmental exposure, is central to microbiome research. These associations can be investigated with differential abundance analysis (DAA), which in its simplest form compares the abundances of microbial taxa between two experimental groups, e.g., subjects with and without the disease in question. Despite this seemingly simple goal, performing DAA reliably has proven to be challenging due to some peculiar statistical properties of microbiome data. Indicative of those challenges, numerous DAA methods developed in recent years tend to yield remarkably differing results [1, 2]. For instance, while one method may detect hundreds of differentially abundant taxa in a particular dataset, another method may detect none [1]. Until now, no consensus has emerged on the best-performing DAA method.

The discrepancy in results obtained from different DAA methods naturally raises the question about which methods provide the most *correct* results. Answering such a question would evidently require knowing the *ground truth,* namely, the true values of taxon-wise differential abundances (DAs), against which the DAA results could be compared. As the ground truth behind any real microbiome dataset is very rarely known, evaluating the correctness of DAA results in practice requires the use of a preset ground truth and *simulated* data.

There is, however, an obvious problem in relying on simulations in that there is no guarantee that the set ground truth or the simulated data would correspond to their real-world equivalents in all relevant aspects. For instance, the assumed abundance differences may not realistically mimic the effects of the studied condition. Furthermore, simulated data may not correspond to count data that would emerge in a real-world experiment under the assumed ground truth. To simulate realistic data, one would need to accurately model different biases that the experimental workflow, consisting of, e.g. DNA extraction and sequencing, introduces to the observed counts. Such biases should include, for instance, taxon-wise biases, addressing the fact that different taxa are detected with varying sensitivities in the experimental workflow [3]. Indicative of the problems in evaluating DAA methods by employing simulated data are the discordant conclusions on methods obtained under different simulation approaches [2, 4–9].

Furthermore, different methods are designed to estimate different types of DA and, consequently, different ground truths. In particular, most currently available taxonomic profiling data are *compositional* [10]. This problem must be accounted for in DAA by what we here call a *normalization strategy*. However, the normalization strategies incorporated in the DAA methods vary. While certain methods *aim* to estimate DA in terms of absolute abundances through employing advanced normalization strategies [11–13], others merely aim to estimate DA in terms of *relative abundances* by employing an equivalent of the simple TSS normalization (counts divided by the library sizes) [14, 15]. Further variation in the targeted ground truths is introduced by the choice between using untransformed or log-transformed counts, i.e. between estimating DA with respect to arithmetic or geometric means. Therefore, due to the ambiguities in the definition of DA, a specific DAA result may not be unambiguously classified as correct or incorrect.

Because of the many problems in evaluating the correctness of DAA results, we decided to focus on the *consistency* and *replicability* of the results. In particular, we study how different methods can replicate statistical significance and the direction (sign) of taxon-wise DA between random partitions of datasets and between different studies. Our goal was to identify methods that can declare a high number of taxa as being associated with the studied condition while being able to accurately reproduce these results in another dataset. Such methods can be considered capable of effectively detecting robust signals in microbiome data. In addition, our approach reveals methods that are not even internally consistent and should therefore be avoided in general. Importantly, we perform all our evaluations by employing datasets from (*N* = 53) real human gut microbiome studies.

Although a few benchmarking studies have examined certain aspects of consistency or replicability on a limited number of datasets [1, 16, 17], a comprehensive benchmarking study focusing on consistency and replicability and employing a large number of datasets is still missing. Furthermore, while we concentrate on evaluating how different DAA methods perform in the basic two-group comparison, we also evaluate the methods in the presence of covariates. Although including covariates is often essential in practice, only a few previous studies have addressed this aspect of DAA, and mostly on simulated datasets [2, 7, 18]. Our study is also the first to evaluate the coverage of the confidence intervals (CI) that different DAA methods provide. Lastly, our comparison includes logistic regression for presence/absence of taxa, which has not been included in previous benchmarking studies.

## Materials and methods

### Analysis frameworks

We extracted 69 datasets from 53 real *human gut microbiome* studies employing 16S rRNA gene sequencing (16S, 23 studies) or shotgun metagenomic sequencing (shotgun, 30 studies) [19, 20]. We included studies that compared groups consisting of healthy and nonhealthy individuals (e.g. colorectal cancer, CRC). We call these groups *control* and *case* groups, respectively. If a single study compared more than two groups (e.g. CRC, adenoma, and healthy), multiple datasets from the study were extracted (e.g. CRC versus healthy and adenoma versus healthy). The most common conditions examined in the included studies were CRC (16 studies), inflammatory bowel disease (9), adenoma (7), obesity (4), type 2 diabetes (4), Clostridioides difficile infection (3), type 1 diabetes (3), autism spectrum disorder (2) and overweight (2). The list and details of the datasets are given in Supplementary Tables A1.1 and A1.2.

**Figure 1:**
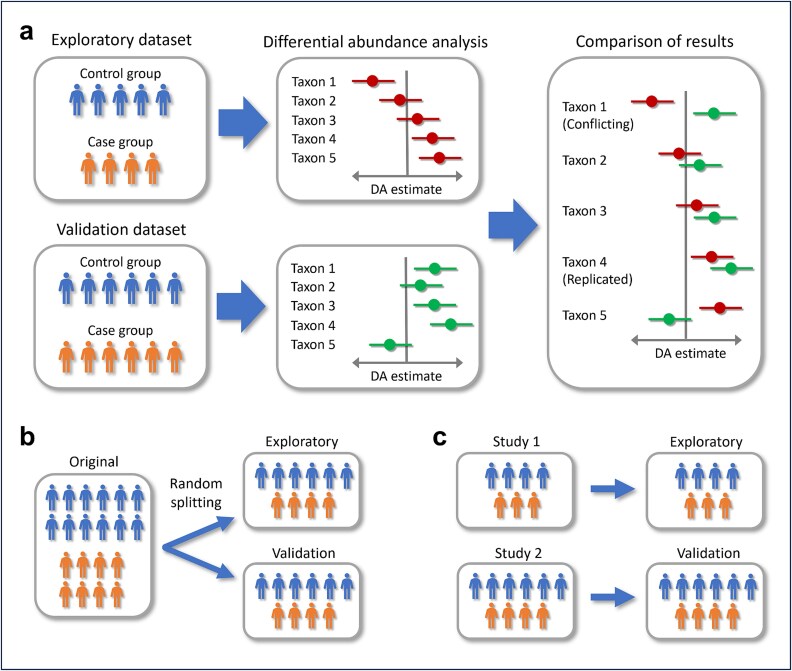
(a) The basic workflow in evaluating replicability and consistency. DAA was performed on exploratory and validation datasets, and the results were compared between them. If the result for a taxon was significant in both exploratory and validation datasets, but the directions were opposite, the results were considered conflicting (Taxon 1). The result for a taxon was considered replicated if it was significant and had the same direction in exploratory and validation datasets (Taxon 4). (b) In the split-data analyses, each exploratory/validation pair of datasets was constructed by randomly splitting an original dataset. (c) In the separate study analyses, datasets from separate studies were used as exploratory and validation datasets. In all subfigures, the individuals belonging to the control and case groups are indicated with blue and orange, respectively.

In all analyses, DAA was first performed in an *exploratory dataset* to search for statistically significant differences in taxon-wise abundances and, subsequently, in the *validation dataset(s)* to validate the results (Fig. 1a). We used two analysis frameworks to study the replication of DAA results both within studies and between studies. In the *split-data analyses* (within study), 285 exploratory-validation pairs of datasets were constructed by randomly splitting 57 original datasets five times into two equal-sized halves (Fig. 1b). These analyses were used to evaluate whether DAA methods perform as consistently as they theoretically should. In the *separate study analyses* (between studies), 50 datasets from separate studies were used as exploratory and/or validation datasets (Fig. 1c). Here, the goal was to investigate how the results provided by each DAA method replicate across studies in practice. In both types of analyses, the required minimum number of subjects in each group (case or control) in each exploratory or validation dataset was 10. A summary of the datasets used in each type of analysis is given in Table 1. Additional details on constructing the pairs of exploratory and validation datasets are given in the Supplementary Appendix.

**Table 1:** Summary of the datasets used in the analyses.

Analysis/sequencing type	Number of datasets	Sample size median (min−max)	Number of taxa median (min−max)	Significant taxa found by at least one method median (min−max)
Split-data analyses				
16S	24 × 5 × 2	57 (21–374)	79 (36–188)	48 (13–104)^1^
Shotgun	33 × 5 × 2	53 (25–255)	201 (91–276)	110 (33–233)^1^
Separate study analysis				
16S	23	112 (32–747)	80 (45–188)	27 (5–81)
Shotgun	27	96 (20–509)	203 (96–260)	44 (9–125)

1 In at least one of the five random splits.

The analyses on 16S and shotgun datasets were performed on the *genus* and *species* level, respectively. Furthermore, following the standard practice in the field, we excluded taxa with prevalence <10%, separately from each exploratory and validation dataset.

### Evaluation metrics

A DAA result for each taxon was defined to be (statistically) significant in the exploratory datasets if the false discovery rate (FDR) adjusted *P*-value was below a chosen *nominal* FDR level α. We note that the true FDR is at (or below) the nominal FDR level α only if a method provides valid *P*-values. We used the standard choice α = 0.05 but repeated the analyses by using α = 0.01, α = 0.10 and α = 0.20. FDR-adjusted *P*-values were calculated by the Benjamini–Hochberg method [21] or by a method integrated into the DAA method (ANCOM-BC2, DESeq2, LDM, ZicoSeq). For each DAA method and within each pair of exploratory and validation datasets, a taxon was called a *candidate taxon* if it was significant in the exploratory dataset and it was present (after the 10% prevalence filtering) in the validation dataset(s) (Taxa 1, 4, and 5 in Fig. 1a). As there were typically under ten candidate taxa, we considered the validation process as testing a few well-formed hypotheses. Therefore, the statistical significance in the validation datasets was defined as *unadjusted P*-value < .05 (for the results with FDR adjustment applied for candidate taxa in the validation datasets, see Supplementary Figs. A20.1 and A20.2). Lastly, the *direction* of a taxon was defined as the sign of its estimated DA (positive, if estimated to be more abundant in the case group). The following metrics were calculated to evaluate the performance of the DAA methods.

#### Percentage of conflicting results

The result for a candidate taxon was defined as *conflicting* if the taxon was significant also in the validation dataset, but the direction was *opposite* to what it was in the exploratory dataset (Taxon 1 in Fig. 1a). A conflicting result was considered a serious error as it would lead to conflicting inferences. Therefore, the *percentage of conflicting results* (*Conflict%*), namely, the percentage of candidate taxa for which a conflicting result was observed, was used as our first main metric for consistency. In the split-data analyses, a conflicting result signifies a false result in either the exploratory or the validation dataset, and we derived an approximate upper limit of α × 0.50 × 0.05 for an ideal Conflict% (for derivation, see the Supplementary Appendix).

#### Replication percentage

The result for a candidate taxon was defined as *replicated* if the taxon was significant also in the validation dataset and it had the *same* direction as in the exploratory dataset (Taxon 4 in Fig. 1a). *Replication percentage* (*Replication%*), namely, the percentage of candidate taxa whose result was replicated in the validation dataset, was our second main metric for consistency. Especially in the split-data analyses, if a method detects any *truly* differentially abundant taxon in an exploratory dataset, it should often be able to detect the same taxon in the validation dataset. Consequently, a low replication percentage may indicate a high FDR.

#### Number of significant taxa

The sensitivity of a given DAA method was measured by the total *number of significant taxa* (number of “hits,” *NHits*) in the exploratory datasets. We note that a high NHits does not necessarily indicate a high power to detect *true* effects as it may also be due to a high number of false positive findings. Nevertheless, when accompanied with low Conflict% and high Replication%, higher NHits can be considered an indication of higher statistical power.

#### Additional metrics: correlation of estimates and overlap percentage of CIs

In addition to the above metrics based on the statistical significance and the direction of DA, we evaluated metrics related to the estimation of the magnitude of DA (i.e. the effect size). The consistency of DA estimates was measured by the Spearman *correlation of the estimates* between each exploratory and the corresponding validation dataset.

Furthermore, we evaluated the *overlap percentage of CIs (CI%)*. For a given taxon, CIs were considered overlapping if there was any overlap between the CIs calculated in the exploratory and the corresponding validation datasets (e.g. the CIs for Taxa 2, 3, and 4 in Fig. 1 were considered overlapping). We examined 83.4% CIs that should ideally overlap (at least) 95% of times in the split-data analyses if the sampling distribution of each DA estimate is approximately normal [22]. CIs were evaluated only for the candidate taxa (with FDR adjusted *P* < .05) with at least 10% prevalence in *both* experimental groups (case and control). LDM, edgeR, and ZicoSeq were excluded from this analysis as they do not provide CIs or standard errors in their output.

### Calculation of the overall values of the evaluation metrics

The overall values of Conflict%, Replication%, and CI% reported in Results were calculated over all candidate taxa in all exploratory datasets. For instance, if 5 out of 8 candidate taxa found in one exploratory dataset replicated and 2 out of 2 candidate taxa found in another exploratory dataset replicated, $\mathrm{Replication}\%=\frac{5+2}{8+2}=0.70=70\%$ (for the details of the separate study analyses, see the Supplementary Appendix). The number of significant taxa (NHits) was the sum of the number of significant taxa in all exploratory datasets. The average correlation of DA estimates was calculated as the hyperbolic tangent transformed mean of the inverse hyperbolic tangent transformed Spearman correlation coefficients.

### Evaluation and ranking of DAA methods

The evaluation and ranking of the DAA methods proceeded as follows. In the split-data analyses, we first examined whether the methods performed adequately according to our criterion for Conflict% and had a high Replication% compared to the other methods. In the separate study analyses, we first considered which methods were among the most consistent methods according to Conflict% and Replication%. Lastly, the methods with adequate/high consistency were ranked by the total number of significant taxa found in the exploratory datasets.

Additionally, we evaluated whether the low consistency of some methods could be justified by their high sensitivity. This was done by comparing the consistency of all methods under constant high sensitivity, that is, when the nominal FDR levels (α) were chosen so that each method detected the same high number of taxa in the exploratory datasets.

### Included DAA methods

We included 14 DAA methods that provided the DA estimate and *P*-value for each taxon and had an up-to-date R implementation available. The collection of methods included recent methods designed specifically for microbial DAA, namely, ANCOM-BC2 [11], corncob [14], fastANCOM [13], LDM [23], LinDA [12], and ZicoSeq [2]. We also included methods that were originally designed for differential expression analysis for RNA-Seq data but which have also been used for microbial DAA, namely, ALDEx2 [24], DESeq2 [25], edgeR [26], limma voom [27, 28] and metagenomeSeq [29].

Additionally, we included two general statistical methods commonly used for DAA in practice [30]. These are the analysis of log-transformed TSS normalized counts with a linear regression model, i.e. *the t*-test when no covariates are included, as implemented in the MaAsLin2 R package [31], and the analysis of (untransformed) TSS normalized counts with a nonparametric method. As the non-parametric method, we chose ordinal regression model (ORM/Wilcoxon), which can be seen as a generalization of the familiar Wilcoxon test in the sense that it can incorporate covariates while giving basically the same *P*-values as the Wilcoxon test in the two-group comparison [32]. Lastly, we included logistic regression analysis of the observed presence/absence (nonzero/zero count) of taxa (LogR).

Except for the LogR approach, the methods can be roughly described in terms of two factors. First, the methods address the compositionality of microbiome data using different normalization strategies. For instance, corncob, LDM, MaAsLin2/*t*-test, and ORM/Wilcoxon (effectively) employ the simple TSS normalization while ALDEx2 (CLR), DESeq2 (RLE), edgeR (TMM), limma-voom (TMM) and metagenomeSeq (CSS) apply more elaborate normalizations to count data. Furthermore, ANCOM-BC2 and LinDA employ an estimated bias correction to the DA estimates, while fastANCOM and ZicoSeq search for nondifferentially abundant taxa and use those as the reference taxa in the analysis.

Second, the methods employ different transformations and statistical models to analyze the (possibly normalized) count data. A linear model/*t*-test with log (or CLR) transformation is employed in ALDEx2, ANCOM-BC2, fastANCOM, limma-voom, LinDA, MaAsLin2/*t*-test, and metagenomeSeq. A linear model is also used in ZicoSeq (with power transformation) and in LDM (with no transformation *and* arcsine-root transformation). Untransformed counts are analyzed with a negative binomial model in DESeq2 and edgeR, and with a beta-binomial model in corncob. Lastly, the ranks of the (TSS normalized) counts are analyzed in ORM/Wilcoxon.

While most of the tested methods had several parameters that could be adjusted by the user, we primarily employed the default settings as this is likely how these methods are applied in practice. For the performance obtained with some other settings, see Supplementary Figs A14.1–A14.4 in the Supplementary Appendix. The list of the compared DAA methods is given in Table 2, and the details of running the methods are presented in the Supplementary Appendix.

**Table 2:** Summary of the compared DAA methods.

Method	Normalization strategy	Performs log (or CLR) transformation	Model	Can incorporate covariates	Provides CIs	Typical run-time
ALDEx2	CLR	x	Linear	x	x	30 s
ANCOM-BC2	Bias correction	x	Linear	x	x	20 s
corncob	TSS		Beta-binomial	x	x	10 s
DESeq2	RLE		Negative binomial	x	x	7 s
edgeR	TMM		Negative binomial	x		0.5 s
fastANCOM	Reference taxa	x	Linear	x	x	0.01 s
LDM	TSS		Linear	x		30 s
limma voom	TMM	x	Linear	x	x	0.07 s
LinDA	Bias correction	x	Linear	x	x	0.2 s
LogR	–		Binary logistic	x	x	0.6 s
MaAsLin2/t-test	TSS	x	Linear	x	x	2 s
metagenomeSeq	CSS	x	Linear		x	0.8 s
ORM/Wilcoxon	TSS		Ordinal (proportional odds)	x	x	3 s
ZicoSeq	Reference taxa		Linear	x		20 s

## Results

### Split-data analyses

**Figure 2:**
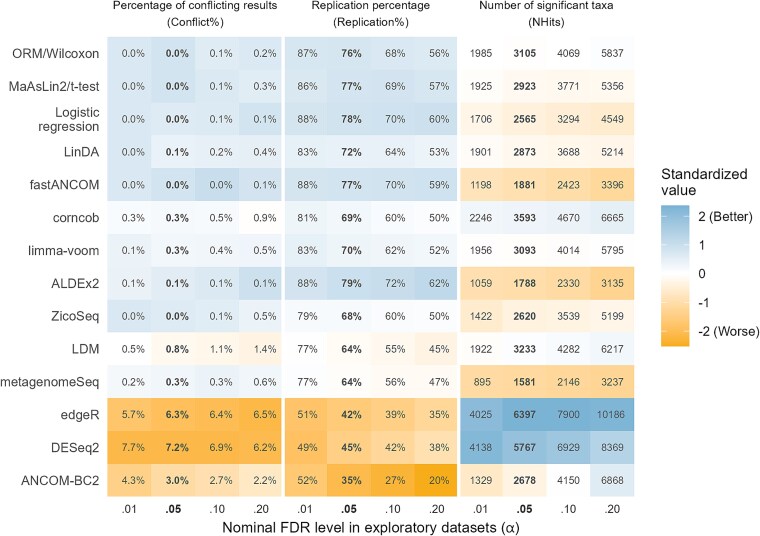
The performance of 14 DAA methods in terms of consistency and sensitivity on 57 randomly split real microbiome datasets. The methods are in rank order based on the mean of the standardized values of the metrics. (Conflict% was square root transformed before the standardization.) Values based on the nominal FDR level *α* = 0.05 are shown in bold. Each original dataset was split five times to form pairs consisting of an exploratory and a validation dataset, thus totaling 285 pairs of datasets. Candidate taxon = A taxon that was significant (FDR-adjusted *P* < *α*) in an exploratory dataset and present in the validation dataset. Conflict% = The percentage of candidate taxa that were significant (*P* < .05) in the validation dataset, but in the opposite direction to that in the exploratory dataset. Replication% = The percentage of candidate taxa that were significant (*P* < .05) in the validation dataset in the same direction as in the exploratory dataset. NHits = The total number of significant (FDR adjusted *P* < *α*) taxa found in the 285 exploratory datasets. A higher NHits can be considered better when it is accompanied by low Conflict% and high Replication%.

**Figure 3:**
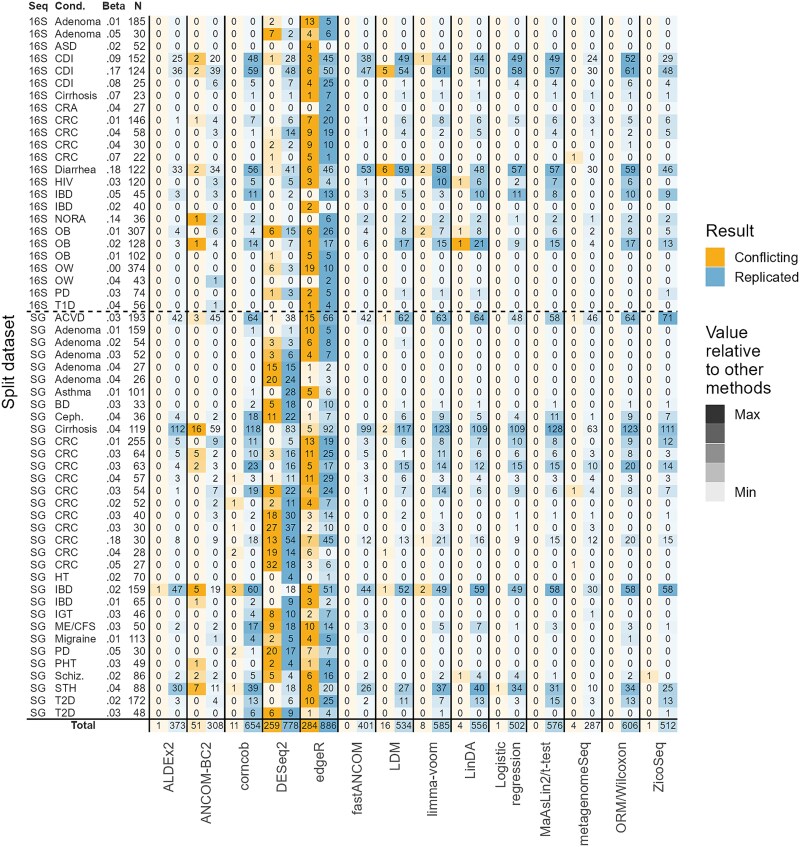
The number of conflicting and replicated results found by 14 DAA methods on 57 randomly split real microbiome datasets. Each original dataset was split to form a pair consisting of an exploratory and a validation dataset. The splitting was performed five times for each original dataset. In each slot is the number of taxa for which a conflicting or replicated result was found in at least one of such pair. Conflicting result = the result for a taxon was significant in the exploratory datasets (FDR adjusted *P* < .05) and validation datasets (*P* < .05) but in opposite directions. Replicated result = the result for a taxon was significant in the exploratory dataset and validation datasets in the same direction. Seq. = sequencing type (16S or SG = shotgun); Cond. = the studied condition; Beta = Beta diversity explained by the experimental group (case/control); *N* = the sample size in a single exploratory or validation dataset. ACVD, atherosclerotic cardiovascular disease; BD, Behcet’s disease; Ceph., cephalosporins; CRA, chronic, treated rheumatoid arthritis; HIV, human immunodeficiency virus; HT, hypertension; IGT, impaired glucose tolerance; ME/CFS, myalgic encephalomyelitis/chronic fatigue syndrome; NASH, nonalcoholic steatohepatitis; NORA, new-onset untreated rheumatoid arthritis; PD, Parkinson's disease; PHT, prehypertension; STH, soil-transmitted helminths.

The overall values of the evaluation metrics are presented in the text and in Fig. 2 (stratified by sample size and sequencing type, see Supplementary Figs A17.1, A18.1 and A19.1, respectively; for the distributions, see Supplementary Figs A4.1–A4.3, A5, and A6 in the Supplementary Appendix). The number of conflicting and replicated results on each randomly split original dataset are given in Fig. 3.

#### Percentage of conflicting results

Seven methods, namely, ALDEx2, fastANCOM, LinDA, LogR, MaAsLin2/*t*-test, ORM/Wilcoxon, and ZicoSeq, performed properly as their Conflict% was mostly below the thresholds for ideal performance, namely, 0.025%, 0.125%, 0.25%, and 0.50% with α = 0.01, 0.05, 0.10, and 0.20, respectively. The performances of corncob (Conflict% = 0.3% at α = 0.05), limma-voom (0.3%), and metagenomeSeq (0.3%) were also tolerable, but for LDM the Conflict% (0.8%) was too high. Most notably, however, ANCOM-BC2 (3.0%) and especially DESeq2 (7.2%) and edgeR (6.3%) had their Conflict% order(s) of magnitude above the acceptable level.

#### Replication percentage

Replication percentages (Replication%) ranged from 35% to almost 80% at α = 0.05. The highest Replication% was observed for ALDEx2 (79%), LogR (78%), fastANCOM (77%), MaAsLin2/*t*-test (77%) and ORM/Wilcoxon (76%). They were followed by LinDA (72%), limma-voom (70%), corncob (69%), ZicoSeq (68%), metagenomeSeq (64%), and LDM (64%). The lowest Replication% was observed for ANCOM-BC2 (35%), DESeq2 (45%), and edgeR (42%).

The high Conflict% and low Replication% observed for ANCOM-BC2, DESeq2, and edgeR indicated that these methods likely provide systematically too low *P*-values. Additional evidence for this was gathered by examining the performance of the methods on 500 (=50 × 10) datasets with randomly permuted group labels (corresponding to a scenario where there are no truly differentially abundant taxa). For instance, instead of the nominal 5%, the percentage of (not multiplicity adjusted) *P*-values < .05 was 18.4% for edgeR and 10.5% for DESeq2 (Supplementary Fig. A7). However, for ANCOM-BC2 this percentage was 3.1%, indicating other reasons for its low consistency.

#### Number of significant taxa

By a large margin the most sensitive methods were edgeR and DESeq2. With the nominal FDR level α = 0.05, they identified a total of 6397 and 5767 significant taxa in the 285 (=57 × 5) exploratory datasets, respectively. They were followed by corncob (NHits = 3593), LDM (3233), ORM/Wilcoxon (3105), limma-voom (3093), MaAsLin2/*t*-test (2923), and LinDA (2873). A little behind these methods were ANCOM-BC2 (2678), ZicoSeq (2620), and LogR (2565). The least sensitive methods were fastANCOM (1881), ALDEx2 (1788), and metagenomeSeq (1581).

While DESeq2 and edgeR identified a high number of taxa (NHits = 5767–6397) with the nominal FDR level α = 0.05, LDM, ZicoSeq, limma-voom, corncob, LinDA, MaAsLin2/*t*-test, and ORM/Wilcoxon detected a number of similar magnitude (NHits around 5000) when α = 0.20 was used. In the latter cases, however, Conflict% was mostly at least an order of magnitude lower, and Replication% was somewhat higher than in the former cases. Moreover, when the nominal FDR levels were chosen so that each method detected exactly 6000 taxa, the highest consistency was achieved by ORM/Wilcoxon, MaAsLin2/*t*-test, and LogR while the lowest was demonstrated by ANCOM-BC2, DESeq2, and edgeR (Supplementary Fig. A3).

#### Correlation of estimates and overlap percentage of CIs

The average Spearman correlation between DA estimates in the exploratory and validation datasets was highest for fastANCOM (0.43), limma-voom (0.43), LinDA (0.43), ALDEx2 (0.43), and MaAsLin2/*t*-test (0.42), followed by ORM/Wilcoxon (0.40), corncob (0.38), ZicoSeq (0.36), LogR (0.36) (Supplementary Fig. A5). The lowest values were observed for metagenomeSeq (0.32), DESeq2 (0.30), edgeR (0.29), LDM (0.28), and ANCOM-BC2 (0.22).

The overlap percentage of CIs (CI%) for candidate taxa was highest for ALDEx2 (93%), LogR (93%), fastANCOM (92%), ORM/Wilcoxon (91%), and MaAsLin2/*t*-test (89%) (Supplementary Fig. A6). It was especially low for DESeq2 (65%) and ANCOM-BC2 (62%).

#### Performance with covariates

The inclusion of 1–3 covariates did not notably decrease the performance of most DAA methods (Supplementary Fig. A8). An exception was DESeq2, whose performance dropped substantially. For instance, its Conflict% was as high as 16.0% and Replication% as low as 25%.

### Separate study analyses

**Figure 4:**
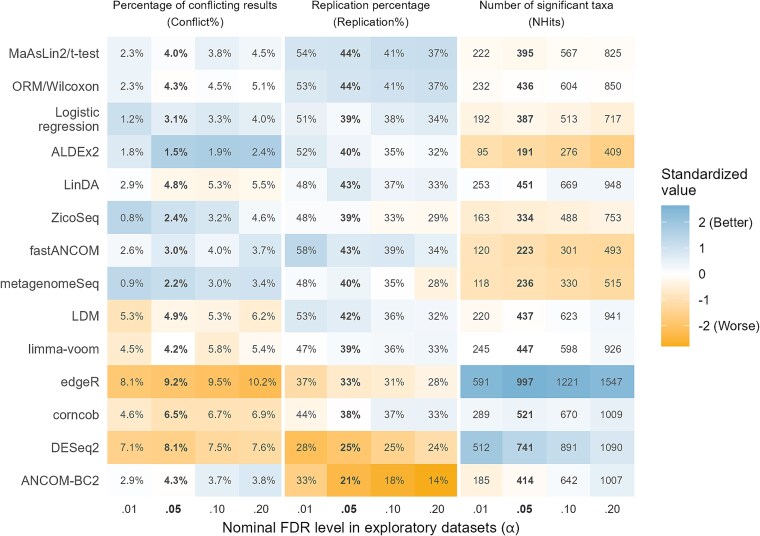
The performance of 14 DAA methods in terms of sensitivity and consistency of results between separate studies. The methods are in rank order based on the mean of the standardized values of the metrics. (Conflict% was square root transformed before the standardization.) Values based on the nominal FDR level *α* = 0.05 are shown in bold. A dataset from one study was used as an exploratory dataset and dataset(s) from other study/studies as the validation dataset(s). Candidate taxon = A taxon that was significant (FDR adjusted *P* < α) in an exploratory dataset and present in a validation dataset. Conflict% = The percentage of candidate taxa that were significant (*P* < .05) in the validation dataset, but in the opposite direction to that in the exploratory dataset. Replication% = The percentage of candidate taxa that were significant (*P* < .05) in the validation dataset in the same direction as in the exploratory dataset. NHits = The total number of significant taxa found in the 37 exploratory datasets. A higher NHits can be considered better when it is accompanied by low Conflict% and high Replication%.

**Figure 5:**
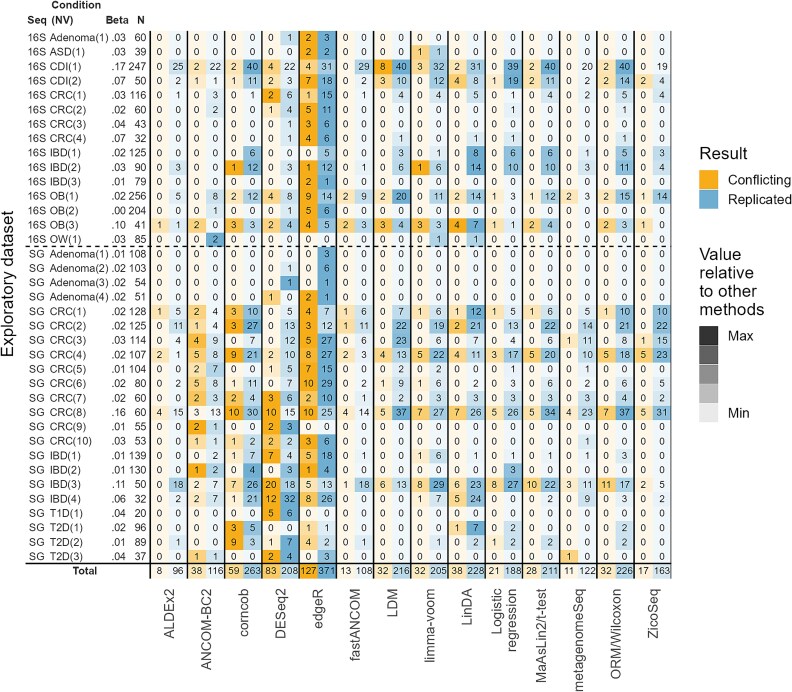
The number of conflicting and replicated results found by 14 DAA methods when datasets from separate studies were used as exploratory and validation datasets. One exploratory dataset may have had multiple validation datasets (indicated by NV). In each slot is the number of taxa for which a conflicting or replicated result was found in at least one of the validation datasets. Conflicting result = the result for a taxon was significant in the exploratory dataset (FDR-adjusted *P* < .05) and validation dataset(s) (*P* < .05) but in opposite directions. Replicated result = the result for a taxon was significant in the exploratory dataset and validation dataset(s) in the same direction. Seq. = sequencing type (16S or SG = shotgun); Condition = the studied condition; Beta = Beta diversity explained by the experimental group (case/control); *N* = the sample size of the exploratory dataset.

The overall values of the evaluation metrics are presented in the text and in Fig. 4 (stratified by sample size and sequencing type, see Supplementary Figs A17.2, A18.2 and A19.2, respectively; for the distributions, see Supplementary Figs A10.1–A10.3, A11, and A12 in the Supplementary Appendix). The number of conflicting and replicated results for each exploratory dataset are given in Fig. 5.

#### Percentage of conflicting results

The lowest Conflict% at the nominal FDR level α = 0.05 was achieved by ALDEx2 (1.5%), followed by metagenomeSeq (2.2%), ZicoSeq (2.4%), fastANCOM (3.0%) and LogR (3.1%). The next lowest values were observed for MaAsLin2/*t*-test (4.0%), limma-voom (4.2%), ORM/Wilcoxon (4.3%), ANCOM-BC2 (4.3%), LinDA (4.8%), and LDM (4.9%). A higher value was observed for corncob (6.5%) and the two highest values for DESeq2 (8.1%) and edgeR (9.2%).

#### Replication percentage

With α = 0.05, the replication percentages (Replication%) varied little (38%–44%) between most of the methods. The methods listed by Replication% from highest to lowest were: MaAsLin2/*t*-test (44%), ORM/Wilcoxon (44%), fastANCOM (43%), LinDA (43%), LDM (42%), ALDEx2 (40%), metagenomeSeq (40%), LogR (39%), limma-voom (39%), ZicoSeq (39%), and corncob (38%). Clearly lower values were observed for edgeR (33%), DESeq2 (25%), and ANCOM-BC2 (21%).

#### Number of significant taxa

The total number of significant taxa found in the 37 exploratory datasets (NHits) with α = 0.05 was clearly highest for edgeR (997), followed by DESeq2 (741). The middle group consisted of corncob (521), LinDA (451), limma-voom (447), LDM (437), ORM/Wilcoxon (436), ANCOM-BC2 (414), MaAsLin2/*t*-test (395), LogR (387) and ZicoSeq (334). The lowest numbers were observed for metagenomeSeq (236), fastANCOM (223), and ALDEx2 (191).

While edgeR detected the highest number of taxa (997) with Conflict% = 9.2% and Replication% = 33% at the nominal FDR level α = 0.05, we note that, for instance, LinDA, LDM, and limma-voom identified almost the same number of taxa (926–948) with lower Conflict% (5.4%–6.2%) and similar Replication% (32%–33%) when α was set at 0.20. Furthermore, when the nominal FDR levels were chosen for each method so that each detected exactly 1000 taxa, the highest consistency was achieved by MaAsLin2/*t*-test, ORM/Wilcoxon, LogR, limma-voom, and LinDA while the lowest consistency was demonstrated by ANCOM-BC2 and DESeq2 (Supplementary Fig. A9).

#### Correlation of estimates and overlap percentage of CIs

The average Spearman correlation between the DA estimates in the exploratory and validation datasets varied little between most methods (Supplementary Fig. A11). It was highest for ORM/Wilcoxon (0.25), MaAsLin2/*t*-test (0.24), LinDA (0.24), limma-voom (0.24), ALDEx2 (0.24), and fastANCOM (0.24). The lowest values were observed for edgeR (0.16), LDM (0.15) and ANCOM-BC2 (0.13).

The overlap percentage of the 83.4% CIs (CI%) varied between 29% and 47%. The highest values were observed for LogR (47%), MaAsLin2 (46%), ORM (44%), and metagenomeSeq (42%), and the lowest for fastANCOM (33%), ALDEx2 (32%), ANCOM-BC2 (32%) and DESeq2 (29%) (Supplementary Fig. A12).

### Summary of the results

#### Split-data analyses

Five methods, ALDEx2, fastANCOM, LogR, MaAsLin2/*t*-test, and ORM/Wilcoxon had adequately low Conflict% while also producing the highest replication percentages. Of these methods, ORM/Wilcoxon and MaAsLin2/*t*-test, followed by LogR, were the most sensitive ones. At the opposite end, the most inconsistent methods by far were ANCOM-BC2, DESeq2, and edgeR. For instance, their Conflict% was order(s) of magnitude higher than what was considered ideal. Moreover, while DESeq2 and edgeR were very sensitive, similar sensitivity *with better consistency* could be reached by other methods (especially ORM/Wilcoxon, MaAsLin2/*t*-test, and LogR) when the nominal FDR level was increased (Supplementary Fig. A3).

As most methods detected most of their significant taxa in larger datasets (sample size *N* > 100), the above overall evaluations were effectively based mainly on the performance on those datasets (Supplementary Fig. A17.1). The most consistent methods did not, however, produce conflicting results even on smaller datasets (*N* < 40).

#### Separate study analyses

Overall, the most consistent methods were ALDEx2, fastANCOM, LogR, MaAsLin2/*t*-test, ORM/Wilcoxon, and ZicoSeq. ORM/Wilcoxon and MaAsLin2/*t*-test, followed by LogR, and ZicoSeq were more sensitive than ALDEx2 and fastANCOM. The least consistent methods were again ANCOM-BC2, DESeq2, and edgeR. There was, however, a strong negative correlation between consistency and sensitivity (Supplementary Fig. A13), and thus a high sensitivity of some methods compensated for their low consistency to some degree. Nevertheless, when comparing the methods under a constant high sensitivity, ORM/Wilcoxon, MaAsLin2/*t*-test, LogR, LinDA, and limma-voom performed slightly better than the other methods in terms of consistency (Supplementary Fig. A9).

## Discussion

We performed a comprehensive investigation of how well microbial DAA results provided by 14 different methods replicate between datasets. We especially studied how different methods can replicate statistical significance and the direction of taxon-wise DA between random partitions of datasets and between different studies. This approach allowed us to identify relatively sensitive methods that perform consistently on datasets from the same study. The identified methods also performed well when considering replication between separate studies. Furthermore, our analyses revealed methods that provide systematically inconsistent results, suggesting caution in their use.

Overall, the best performance was obtained by analyzing TSS normalized counts, i.e. relative abundances, with a nonparametric method (ORM/Wilcoxon), log-transformed TSS normalized counts with the *t*-test/linear regression (MaAsLin2/*t*-test), or the presence/absence of taxa with logistic regression (LogR). These methods performed adequately according to our criteria in the split-data analyses and were among the most consistent ones also in the separate study analyses. Importantly, they were clearly more sensitive than the other consistent methods. These methods were also the most consistent ones when all methods were forced to detect a high constant number of taxa by adjusting the nominal FDR levels. Moreover, they performed above average when considering the correlation of DA estimates and the overlap percentage of CIs.

At the opposite end, ANCOM-BC2, DESeq2, and edgeR were found to be highly inconsistent. For instance, in the split-data analyses, these methods provided at least an order of magnitude more conflicting results than what was considered acceptable. Our finding is in line with some other studies where correspondingly poor performance of DESeq2 and especially edgeR has been observed in the form of high error rates in group label shuffling on real datasets [1, 2, 16]. Moreover, when covariates were included, the performance of DESeq2 dropped further and the percentages of conflicting and replicated results were almost of the same order of magnitude (Supplementary Fig. A8). Lastly, while DESeq2 and especially edgeR were clearly the most sensitive methods, we observed that a similar sensitivity could be achieved more reliably by other methods by using a higher nominal FDR level.

Of the rest of the methods, ALDEx2 and fastANCOM showed good consistency but were notably less sensitive compared to MaAsLin2/*t*-test and ORM/Wilcoxon. This finding aligns with previous studies that found ALDEx2 to be conservative [1, 2, 16, 33], yet consistently performing [1, 16]. Furthermore, LinDA and limma-voom had little deficiencies in consistency while being acceptably sensitive. Lastly, corncob, LDM, metagenomeSeq, and ZicoSeq compromised a little in consistency and/or sensitivity.

An important observation in our investigation was that the best performance was obtained by the elementary methods that are not specifically designed for (microbial) DAA. It therefore seems that applying advanced strategies to address the compositionality of microbiome data and/or employing complex statistical models reduces either the consistency or sensitivity achieved in DAA. We interpret this to indicate that it may not be generally beneficial to employ complex statistical modeling techniques in DAA. Especially considering the highly inconsistent performance of DESeq2 and edgeR, the employment of the negative binomial model may not be advisable in DAA (for pure negative binomial model, see Supplementary Figs A14.1–A14.4).

Nevertheless, it is well-known that analyzing simple TSS normalized counts can lead to spurious results if the total absolute abundances differ systematically between the experimental groups [5, 9, 34, 35]. Therefore, despite the possible loss in consistency and/or sensitivity, complex normalization strategies may be needed in DAA to avoid such spurious findings. To investigate this, we performed DAA on a real dataset from a study on mice where the absolute microbial abundances were *measured* to be clearly higher in one group [36]. We observed that the methods employing TSS normalization estimated the sign of the “true” DA (based on the measured absolute abundances) comparably to the other methods (Supplementary Fig. A15). Moreover, in our main analyses the consistency of MaAsLin2/*t*-test and ORM/Wilcoxon was comparable to other methods on all datasets (Figs 3 and 5), and their *P*-values changed little when TSS normalization was replaced by more advanced normalization methods, such as CSS, GMPR [37], TMM or Wrench [38] (Supplementary Fig. A16). Consequently, while this examination was very limited and our analyses may not include datasets with very large systematic differences in total absolute abundances, we gathered some indirect evidence indicating that sophisticated normalization methods may offer little advantage over simple TSS normalization in many typical studies on human gut microbiome.

While the consistency of several DAA methods was appropriate in the split-data analyses, a general observation was that DAA results were generally substantially less consistent in the separate study analyses. This indicates that any between-study differences in DAA depend largely on actual differences between the study populations and/or differences in the experimental workflows. The possible low replicability of DAA results across studies is thus not necessarily due to any faulty performance of the DAA method. Nevertheless, while the consistency was lower in the separate study analyses, the more consistent methods produced 10–20 times more replicated findings than conflicting results.

CIs have not been investigated in previous benchmarking studies on DAA. We found that at least some methods (ALDEx2, fastANCOM, LogR, MaAsLin2/*t*-test, and ORM/Wilcoxon) provided relatively consistent intervals in the split-data analyses. We note, however, that a high overlap percentage does not necessarily indicate accurate estimates but may also reflect extensively wide CIs. Moreover, apart from DESeq2, adding covariates in the analysis did not essentially alter the performance of the tested DAA methods. Additionally, the run-time of each tested method was mostly under one minute and should thus not be an issue in practice (Table 2, Supplentary Fig. A2).

An interesting finding was that when simply analyzing the presence/absence of taxa (LogR), the sensitivity was only around 15% lower than that of MaAsLin2/*t*-test and ORM/Wilcoxon. These observations are in line with two recent studies [39, 40], and they suggest that a substantial amount of relevant information in microbial count data may lie merely in the observed presence/absence of taxa.

It has been reported that the general performance of DAA methods may depend on data characteristics [9] and that the consistency of any method may depend strongly on the dataset [16]. It can therefore be considered a limitation of our study that we merely investigated the methods’ overall performance. However, when replication of DAA results is considered, it would be especially relevant to use the same DAA method in all compared studies. It is therefore important to identify methods that work reasonably well on most datasets.

Due to data availability reasons, we employed only datasets from human gut microbiome studies. Moreover, we always filtered out taxa with prevalence <10% and did not consider rarefying data. We cannot guarantee how well our findings would apply on other types of data. Furthermore, while we used datasets from a reasonably large number (*N* = 53) of studies, the total number of candidate taxa in the separate data analyses was relatively low (167–785) and most candidate taxa were from a few exploratory datasets. The effect of random variation was thus considerable on our findings in the separate study analyses. Those findings should therefore be seen as only rough indications for the expected replicability of DAA results between studies.

**Key Points**

- We benchmarked 14 differential abundance analysis methods by examining the consistency and replicability of their results along with their sensitivity.
- We employed 69 real datasets from 53 microbiome studies.
- The highest consistency with good sensitivity was obtained by analyzing relative abundances or presence/absence with elementary methods (nonparametric test, linear regression/*t*-test, logistic regression).
- Some widely used methods were found to perform highly inconsistently.
- A higher sensitivity should be aimed for by using a consistent method with a higher significance level.

## Supplementary Material

Appendix_Pelto_BiB_final_bbaf130

## Data Availability

The curated datasets and codes supporting the conclusions of this article are available in Zenodo https://doi.org/10.5281/zenodo.15047338. The original datasets employed in this study are available in the MicrobiomeHD database [19] or in the curatedMetagenomicData (version 3.10.0) R package [20]. All data curation and analyses were performed in R 4.2.3 [41].
