# Supplementary material for: Elementary methods provide more replicable results in microbial differential abundance analysis
Source: Brief Bioinform. 2025 Mar 26;26(2):bbaf130. doi: 10.1093/bib/bbaf130 (PMC11937625; doi:10.1093/bib/bbaf130)
Supplement: Appendix_Pelto_BiB_final_bbaf130 [file appendix_pelto_bib_final_bbaf130.docx]

**Appendix of “Elementary methods provide more replicable results in microbial differential abundance analysis”**

**The datasets employed in the analyses**

Condition: the studied condition; Split: included in the split-data analyses; Separate: included in the separate study analyses; Covariates: of age, sex, and BMI, those that were provided with the dataset; N(Control/Case): the sample size (number of participants) of control/case group; Avg. reads: geometric mean of total read counts (i.e. sequencing depths or library sizes).

| **Study** | **Type** | **Condition** | **Split** | **Separate** | **Covariates** | **N (Control)** | **N (Case)** | **Avg. reads** |
| --- | --- | --- | --- | --- | --- | --- | --- | --- |
| [1] | 16S | T1D | x |  | Age, Sex | 55 | 57 | 8 706 |
| [2] | 16S | Adenoma | x | x | Age, Sex, BMI | 172 | 198 | 9 552 |
| [2] | 16S | CRC | x | x | Age, Sex, BMI | 172 | 120 | 10 142 |
| [3] | 16S | CRC | x | x |  | 22 | 21 | 1 220 |
| [4] | 16S | IBD |  | x |  | 16 | 146 | 9 282 |
| [5] | 16S | Obesity | x | x | Age, BMI | 428 | 185 | 25 671 |
| [5] | 16S | Overweight | x | x | Age, BMI | 428 | 319 | 25 713 |
| [6] | 16S | ASD |  | x |  | 20 | 19 | 1 414 |
| [7] | 16S | IBD |  | x | Sex | 18 | 107 | 1 045 |
| [8] | 16S | HIV | x |  | Sex | 34 | 205 | 10 074 |
| [9] | 16S | IBD | x | x | Age, Sex | 24 | 66 | 1 884 |
| [10] | 16S | Parkinson | x |  |  | 74 | 74 | 2 656 |
| [11] | 16S | CRA | x |  |  | 28 | 26 | 2 529 |
| [11] | 16S | NORA | x |  |  | 28 | 44 | 2 393 |
| [12] | 16S | CDI | x | x | Age, Sex | 154 | 93 | 4 605 |
| [12] | 16S | Diarrhea | x |  | Age, Sex | 154 | 89 | 4 687 |
| [13] | 16S | EDD (CDI) | x | x |  | 82 | 222 | 2 667 |
| [14] | 16S | ASD | x | x | Sex | 44 | 59 | 5 279 |
| [15] | 16S | Obesity | x | x |  | 61 | 195 | 2 159 |
| [15] | 16S | Overweight | x | x |  | 61 | 24 | 2 565 |
| [16] | 16S | CDI | x | x |  | 25 | 25 | 3 290 |
| [17] | 16S | CRC |  | x |  | 18 | 14 | 241 |
| [18] | 16S | IBD | x | x | Age, Sex | 35 | 44 | 1 332 |
| [19] | 16S | Adenoma | x | x | Age, Sex | 30 | 30 | 52 774 |
| [19] | 16S | CRC | x | x | Age, Sex | 30 | 30 | 57 504 |
| [20] | 16S | CRC | x | x | Age, Sex, BMI | 75 | 41 | 112 207 |
| [21] | 16S | Cirrhosis | x |  |  | 23 | 23 | 719 |
| [22] | 16S | Obesity |  | x | Age, Sex | 16 | 25 | 9 864 |
| [23] | 16S | Obesity | x | x | Sex | 100 | 104 | 3 548 |

**Table A1.1** The 16S datasets used in this benchmarking study

| **Study** | **Type** | **Condition** | **Split** | **Separate** | **Covariates** | **N (Control)** | **N (Case)** | **Avg. reads** |
| --- | --- | --- | --- | --- | --- | --- | --- | --- |
| [24] | Shotgun | Parkinson | x |  |  | 28 | 31 | 26 802 551 |
| [25] | Shotgun | Adenoma | x | x | Age, Sex, BMI | 61 | 47 | 52 045 350 |
| [25] | Shotgun | CRC | x | x | Age, Sex, BMI | 61 | 46 | 52 597 788 |
| [26] | Shotgun | CRC | x | x | Age, Sex, BMI | 30 | 30 | 8 636 870 |
| [27] | Shotgun | IBD |  | x |  | 12 | 20 | 27 617 834 |
| [28] | Shotgun | Adenoma | x | x | Age, Sex, BMI | 28 | 26 | 5 128 558 |
| [28] | Shotgun | CRC | x | x | Age, Sex, BMI | 28 | 27 | 5 559 781 |
| [29] | Shotgun | T1D |  | x | Age, Sex, BMI | 10 | 10 | 44 414 623 |
| [30] | Shotgun | IBD |  | x | Age | 38 | 12 | 3 714 882 |
| [31] | Shotgun | ACVD | x |  | Sex | 171 | 214 | 52 656 199 |
| [32] | Shotgun | IGT | x |  | Age, BMI | 43 | 49 | 26 104 542 |
| [32] | Shotgun | T2D | x | x | Age, BMI | 43 | 53 | 27 820 530 |
| [33] | Shotgun | IBD |  | x | BMI | 10 | 129 | 69 215 316 |
| [33] | Shotgun | T1D |  | x |  | 10 | 31 | 53 985 803 |
| [33] | Shotgun | T2D |  | x |  | 10 | 79 | 55 798 662 |
| [34] | Shotgun | Hypertension | x |  |  | 41 | 99 | 44 498 422 |
| [34] | Shotgun | Pre-hypertension | x |  |  | 41 | 56 | 44 178 298 |
| [35] | Shotgun | ME/CFS | x |  | Sex, BMI | 50 | 50 | 54 649 832 |
| [36] | Shotgun | IBD | x | x | Sex, BMI | 236 | 81 | 51 814 434 |
| [37] | Shotgun | T2D | x | x | Sex, BMI | 174 | 170 | 38 298 897 |
| [38] | Shotgun | Cirrhosis | x |  | Age, Sex, BMI | 114 | 123 | 44 482 671 |
| [39] | Shotgun | Cephalosporins | x |  | Age, Sex, BMI | 36 | 36 | 125 324 066 |
| [40] | Shotgun | STH | x |  | Age, Sex, BMI | 86 | 89 | 17 255 200 |
| [41] | Shotgun | T2D |  | x | Age, Sex, BMI | 18 | 19 | 44 929 627 |
| [42] | Shotgun | IBD | x | x | Age, Sex | 27 | 103 | 19 628 426 |
| [43] | Shotgun | Adenoma | x | x | Age, Sex, BMI | 24 | 27 | 89 916 338 |
| [43] | Shotgun | CRC | x | x | Age, Sex, BMI | 28 | 32 | 39 538 771 |
| [43] | Shotgun | CRC | x | x | Age, Sex, BMI | 40 | 40 | 42 904 588 |
| [43] | Shotgun | CRC | x | x | Age, Sex, BMI | 24 | 29 | 95 517 110 |
| [44] | Shotgun | CRC | x | x | Age, Sex, BMI | 52 | 52 | 63 755 391 |
| [45] | Shotgun | CRC | x | x | Age, Sex, BMI | 65 | 60 | 48 601 165 |
| [46] | Shotgun | Asthma | x |  | Age, BMI | 177 | 24 | 72 321 657 |
| [46] | Shotgun | Migraine | x |  | Age, BMI | 177 | 49 | 72 195 837 |
| [47] | Shotgun | Adenoma | x | x | Age, Sex, BMI | 251 | 67 | 42 606 059 |
| [47] | Shotgun | CRC | x | x | Age, Sex, BMI | 251 | 258 | 41 899 750 |
| [48] | Shotgun | BD | x |  | Age, Sex, BMI | 45 | 20 | 41 731 548 |
| [49] | Shotgun | CRC | x | x | Age, Sex, BMI | 54 | 74 | 55 154 620 |
| [20] | Shotgun | Adenoma | x | x | Age, Sex, BMI | 61 | 42 | 54 303 740 |
| [20] | Shotgun | CRC | x | x | Age, Sex, BMI | 61 | 53 | 55 276 139 |
| [50] | Shotgun | Schizophrenia | x |  | Age, Sex, BMI | 81 | 90 | 73 408 097 |

**Table A1.2** The shotgun datasets used in this benchmarking study

**Additional details on the analysis frameworks**

*Construction of the pairs of datasets in the split-data analyses*

In the split-data analyses, each exploratory-validation pair of datasets was constructed by randomly splitting an original dataset into two equal sized halves. Within each pair, one of the halves was randomly chosen as the exploratory dataset and the other one as the validation dataset. The splitting was done stratified by the case/control status (see Figure 1b). Only original datasets with at least 20 samples per group were used. This criterion was fulfilled by 57 datasets (from 43 studies). To increase the number of dataset pairs and thus to decrease the randomness in the results, we performed the splitting five times for each dataset. This resulted in 285 (57 x 5) pairs of datasets.

*Construction of the pairs of datasets in the separate study analyses*

In the separate study analyses, each exploratory-validation pair was made up from datasets from different studies which examined the same condition and had the same sequencing type. Within each pair, the dataset with the smaller sample size was set as the exploratory dataset because the results from a smaller study are more likely to replicate in the larger study than vice versa. We included only datasets with at least 10 samples per group (and with at least one taxon detected by at least one method with FDR level .05). This yielded the inclusion of 50 datasets (23 16S and 27 shotgun datasets), of which 37 (15 16S and 22 shotgun datasets) were used as exploratory datasets. In the cases of several pairs of datasets having the same exploratory dataset, the overall values of Conflict% and Replication% were calculated so that each taxon in each exploratory dataset received an equal weight in the calculations (see below for details).

*Analyses with covariates*

We carried out the split-data analyses also by including covariates in DAA. As age, BMI, and sex are typical covariates in human microbiome studies and at least some of them were provided with most of the original datasets, we included the available ones of these three covariates in DAA. There were 45 original datasets that provided age, sex or BMI and were eligible to the split-data analyses. Covariates included in each dataset are given in Tables A1.1 and A1.2. As metganomeSeq did not provide the possibility to include covariates, it was excluded from this analysis.

Age and BMI were treated as continuous variables and standardized before DAA. If at most 10% of the values of any covariate were missing, they were imputed by the group-wise median (age and BMI) or mode (sex) of the covariate. If >10% of the values were missing, the covariate was not included in DAA.

As the covariates were not chosen by subject matter considerations but merely by their availability and by their common use in microbiome studies, the goal of these analyses was not to evaluate how accurately the DAA methods can control for the effect of confounding variables. Instead, the goal was to evaluate how robustly different DAA methods perform when a more complex analysis than a mere two-group comparison is performed. Especially, we compared how the replication performance of each DAA method changed when covariates were included.

**Derivation of the acceptable level for the percentage of conflicting results in the split-data analyses**

For simplicity, we assume below that significance level α = .05 is used in the exploratory datasets.

As statistical significance was defined in the exploratory datasets as (FDR adjusted p =) q < .05, a properly performing method should control the false discovery rate (FDR) at level .05. Consequently, at most 5% of the *candidate* taxa can be allowed to have incorrectly estimated the sign of DA in an exploratory dataset. Assuming that for most taxa the “true” DA is approximately zero, the “true” DA is likely close to zero also for the findings with incorrectly estimated sign. Therefore, as significance is based on unadjusted p-values < .05 in the validation datasets, a proper DAA method should provide a significant result for at most 5% of these findings in the validation dataset. Furthermore, half of these findings should be estimated to have the opposite direction to that in the exploratory dataset. Consequently, we obtain an approximation for an upper limit of acceptable Conflict%: .05 × .05 × .50 = .00125 or .125%. Thus, Conflict% < .125% can be interpreted to indicate the method providing proper p and q values.

**Calculation of 83.4% confidence intervals**

If a method provided confidence intervals in its output, they were used in our analyses. If a method provided only standard errors (SE), the 83.4% confidence intervals (CI) were calculated as follows. CI = $\hat{\beta}$ +/- t(.917)_df_ × SE, where $\hat{\beta}$ is the DA estimate and t(.917)_df_ is the 1 - (1 – .834) / 2 = .917 quantile of the t-statistic with df degrees of freedom. The degrees of freedom were chosen so that the confidence intervals matched the p-values provided by the method.

**Details on the calculation of Conflict%, Replication% and CI% in the separate study analyses**

In the separate study analyses, in case there were multiple pairs of datasets with the same exploratory dataset, the results in the validation datasets were weighted so that each candidate taxon in each exploratory dataset received an equal weight in the calculations. The calculation is best illustrated with an example. We use Replication% here as an example but values for Conflict% and CI% were calculated in a similar manner.

Assume an exploratory dataset E1 had three validation datasets V1a, V1b and V1c and assume that four taxa (Taxon 1, Taxon 2, Taxon 3, and Taxon 4) were significant in E1. Let us further assume that Taxon 1 was absent in all validation datasets, Taxon 2 was present in V1a and V1b but replicated only in V1a, Taxon 3 was present in V1b and V1c but replicated in neither of them, and Taxon 4 was present in all validation datasets but replicated only in V1a and V1c. Finally, assume that an exploratory dataset E2 had validation datasets V2a and V2b, and that only Taxon 4 was significant in E2 and present only in V2a where it replicated.

There were thus three candidate taxa from E1 (Taxon 2, Taxon 3 and Taxon 4; note that Taxon 1 was absent in V1a, V1b and V1c and was therefore not considered as a candidate taxon) and one candidate taxon from E2 (Taxon 4). In E1 Taxon 2 was 1/2 replicated, Taxon 3 was not replicated, and Taxon 4 was 2/3 replicated. In E2 Taxon 4 was fully replicated as it was replicated in the datasets (only V2a) where it was present. Now Replication% = ([1/2 + 0 + 2/3] + [1]) / (3 + 1) = 54.2%.

**Details on running the DAA methods**

The DAA methods were mostly run with the default settings. However, if a method provided a filter for taxa with too low prevalence, filtering was not used as we had removed taxa with prevalence < 10%. All other exceptions from the default settings are detailed below.

*ALDEx2*

Functions in the R package *ALDEx2* were used. First, CLR transformed Monte Carlo samples from the Dirichlet distribution were generated using the *aldex.clr* function. The default number (n = 128) of Monte Carlo samples were generated. The function *aldex.glm* was then run to perform DAA. Confidence intervals were calculated based on the standard errors provided by *aldex.glm*. Additionally, we ran ALDEx2 using the default *aldex* function (where covariates cannot be included). The t-test-based p-values were very similar to those provided by *aldex.glm* (data not shown). Instead, the results based on Wilcoxon test (of ALDEx2) (ALDEx2-Wilcox in Figures A14.1-A14.4) were slightly different from those based on the *aldex.glm*. Furthermore, by setting the gamma parameter to a non-null value (gamma = 0.5) in the *aldex* function, we were able to incorporate scale uncertainty into ALDEx2 [51]. This version of ALDEx2 was even less sensitive than the standard version (ALDEx2-scale in Figures A14.1-A14.4).

*ANCOM-BC2*

The function *ancombc2* from the R package *ANCOMBC* was used to perform DAA. Importantly, *the taxa that did not pass the sensitivity analysis for zeros were never considered significant*, as suggested by the authors of ANCOM-BC2. Furthermore, *ancombc2* function was run separately for the p values (with p_adj_method = 'none' and alpha = .05) and for the FDR adjusted p values on each FDR level (by setting p_adj_method = 'BH' and alpha = .01, .05, .10 or .20). This was done to the sensitivity analysis for zeros to work properly. Otherwise the *ancombc2* function was used with the default settings, e.g. the detection of structural zeros was not implemented (struc_zero = FALSE).

*corncob*

The function *differentialTest* from the R package *corncob* was used to perform DAA. The dispersion parameter was *not* allowed to vary between groups (phi.formula = ~ 1 and phi.formula_null = ~ 1). This choice was made as we observed that the performance of *corncob* dropped drastically when dispersion was allowed vary between the groups (corncob-UEV in Figures A14.1-A14.4). We used p-values based on the likelihood ratio test (test = “LRT”) as they were found to give a little better results (higher sensitivity) compared to results based on Wald p-values (data not shown). The confidence intervals were based on the Wald’s approximation, however, and they did not therefore match exactly the p-values based on LRT.

*DESeq2*

Functions in the R package *DESeq2* were used. First, a *DESeqDataSet* object was created from the count matrix by using *DEseqDatasetFromMatrix.* Next, size factors were estimated using *EstimateSizeFactorsForMatrix* (with type = “poscounts”). The results based on the Wald test were then calculated with the function *DESeq*. In the exploratory datasets, we used the adjusted p-values provided by DESeq (instead of performing the Benjamini-Hochberg correction for the raw p-values). Additionally, we calculated results based on the likelihood ratio test (*DESeq* function with test = “LRT” and the model without the group variable as the reduced model). With the likelihood ratio test, DESeq2 had lower error rates (without covariates) but was also clearly less sensitive (DESeq2-LRT in Figures A14.1-A14.4). Furthermore, we tried DESeq2 with GMPR normalized counts (*GMPR* function from *GUniFrac* package). The results, however, were not better than with the default size factors. (Data not shown).

*edgeR*

Functions in the R package *edgeR* were used. First, *DGEList* object was created using the *DGEList* function. Next, the default TMM normalization factors were calculated with function *calcNormFactors*. Then dispersions were estimated with the *estimateDisp* function. Finally, the results based on the quasi-likelihood test were calculated using the *glmQLFTest function*.

*fastANCOM*

DAA was performed using the *fastANCOM* function (from package *fastANCOM*) with the default settings (pseudo = 0.5, sig = 0.05 and ref.rate = .05, struc_zero = FALSE). Statistical significance was based on the p-values and not on the fraction of rejected log-ratio tests (*REJECT* value) provided by *fastANCOM*. This choice was made so that the performance of fastANCOM could be compared to that of the other methods.

*LDM*

DAA was performed using function *ldm* (from package *LDM*) with the default settings. The q values provided by LDM were used as adjusted p-values in the exploratory datasets. The p and q values based on the omnibus test combing the results with non-transformed and arcsine-root-transformed abundances were used (p.otu.omni and q.otu.omni). We also run LDM with CLR transformed counts (comp.anal = TRUE) as suggested recently [52] but this did not improve the performance of LDM (LDM-CLR in Figures A14.1-A14.4).

*limma-voom*

Functions from packages *edgeR* and *limma* were used. First, a *DGEList* object was created from count matrix using the *DGEList* function. Then TMM normalization factors were calculated using function *calcNormFactors*. Next, the mean-variance relationship and thus the weights for the observations were calculated using the *voom* function. A linear model was then fitted for each taxon using the lmFit function. Lastly, the final results based on the empirical Bayes moderated standard errors were obtained by applying the function *eBayes*.

*LinDA*

DAA was performed using the function *linda* from package *LinDA* with the default settings (adaptive = TRUE, pseudo.cnt = 0.5). As the zero-handling approach may have varied between exploratory and validation datasets when the default “Adaptive” setting was used, we tried LinDA also with the pseudo-count and imputation approaches available in the *linda* function. Using these alternative options had very little effect on the results, however (Data not shown).

*LogR (Logistic regression for presence/absence of taxa)*

The count data were first transformed so that non-zero counts (present taxa) were replaced by 1 and zero counts (absent taxa) were left as zeros. The DAA for each taxon was then performed using Firth type logistic regression [53]. This was implemented using the *logistf* function in the *logistf* R package. We used this robust version of logistic regression as the sample size was rather small (N = 20) in some cases and, furthermore, in some cases the prevalence of some taxa was zero in one of the groups.

*MaAsLin2/t-test*

DAA was performed using function *Maaslin2* in the R package *Maaslin2*. We used the default approach, i.e. a linear model for log transformed TSS normalized counts (analysis_method = “LM”, transform = “LOG” and normalization = “TSS”). Additionally, we tried MaAsLin2 with arcsine-square root transformed relative counts (transform = AST) as in [54], but this only dropped the number of replicated taxa detected by it (MaAsLin2-AST in Figures A14.1 - A14.4). We also tried MaAsLin2 with CLR, CSS and TMM normalized counts (normalization = “CLR”, “CSS” or “TMM”, respectively) but using these alternative normalizations did not improve its performance (MaAsLin2-CLR, MaAsLin2-CSS and MaAsLin2-TMM in Figures A14.1 – A14.4).

*metagenomeSeq*

Functions in R package *metagenomeSeq* were used. First, an *MRexperiment* object was created from the count matrix by using the function *newMRexperiment*. Next, CSS normalization factors were calculated using the function *cumNorm* (with parameter p = 0.5). DAA was then performed using the *fitFeatureModel* function.

*NegBin (Negative binomial regression)*

We included in the additional analyses also pure negative binomial regression (Figures A14.1 – A14.4). The analyses were performed using function *glmmTMB* (with family = nbinom2) in the R package *glmmTMB*. We used log(library size) as an offset term to effectively achieve TSS normalization.

*ORM/Wilcoxon (Ordinal regression model)*

An ordinal regression model was used to analyze TSS normalized counts for each taxon. The analyses were performed using the function *orm* in the R package *rms*. We used p-values based on the score test as they were closest to p-values from the Wilcoxon test. We also tried p-values based on the likelihood ratio and Wald tests. The former were slightly anti-conservative when the prevalence of a taxon was zero in one group and the latter were not sensible in such cases (Data not shown). The confidence intervals were based on the Wald’s approximation, however, and did not therefore match exactly the p-values based on the score test. Additionally, we used ORM/Wilcoxon with GMPR and Wrench normalized counts (Figures A14.1 – A14.4).

*radEmu*

DAA was performed using the *emuFit* function from the *radEmu* package. We extracted p values based on Wald tests instead of the default score tests (return_wald_p = T, run_score_tests = F) as the score tests took an excessively long time to converge. We did not include radEmu in the main text of this study because it was introduced in a paper that was available only as a pre-print [55].

*ZicoSeq*

DAA was performed using the *ZicoSeq* function (from the *GUniFrac* package) with the default settings (e.g. is.winsor = TRUE, is.post.sample = TRUE). Especially, we employed the default square root link function [link.func = list(function(x) sign(x) * (abs(x))^0.5)]. The number of permutations was, however, set to 999 (perm.no = 999), instead of the default 99, to obtain more reliable p values.

There were also two very recent DAA methods that we originally planned to be included in this study, i.e. DACOMP [56] and LOCOM [57]. They were eventually excluded, however, as they did not always provide all the required quantities (p-value and DA estimate) and their performance was thus difficult to compare to the other methods.

**
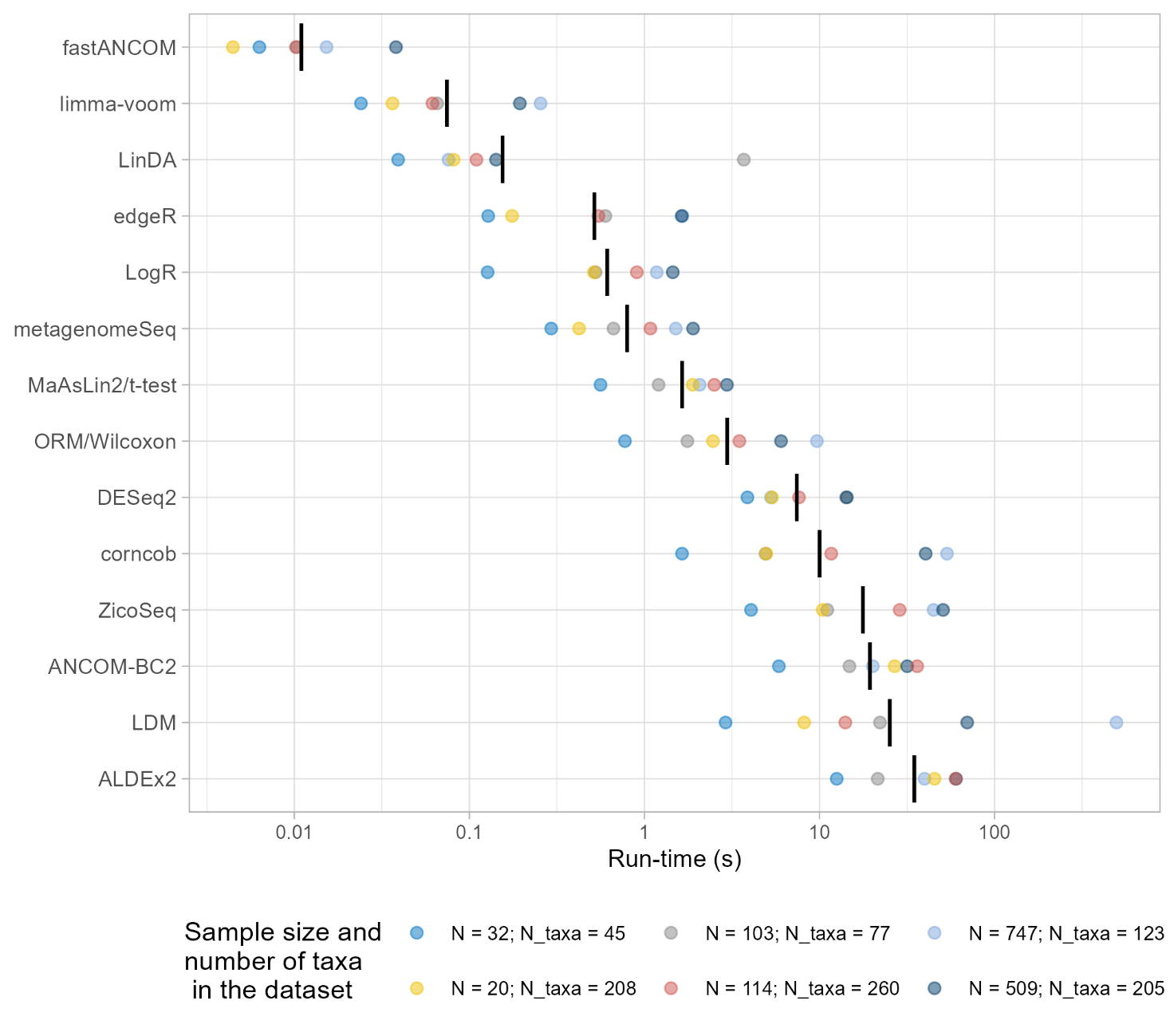
**

**Figure A2** The run-times of the 14 compared DAA methods on six microbiome datasets with varying numbers of samples and taxa (shown in the legend). The black vertical lines are the geometric means of the run-times (given in Table 2). The methods were run on a standard laptop (Intel(R) Core(TM) i7-8565U CPU @ 1.80GHz 1.99 GHz, 16GB RAM, 64bit, Windows 11).

**
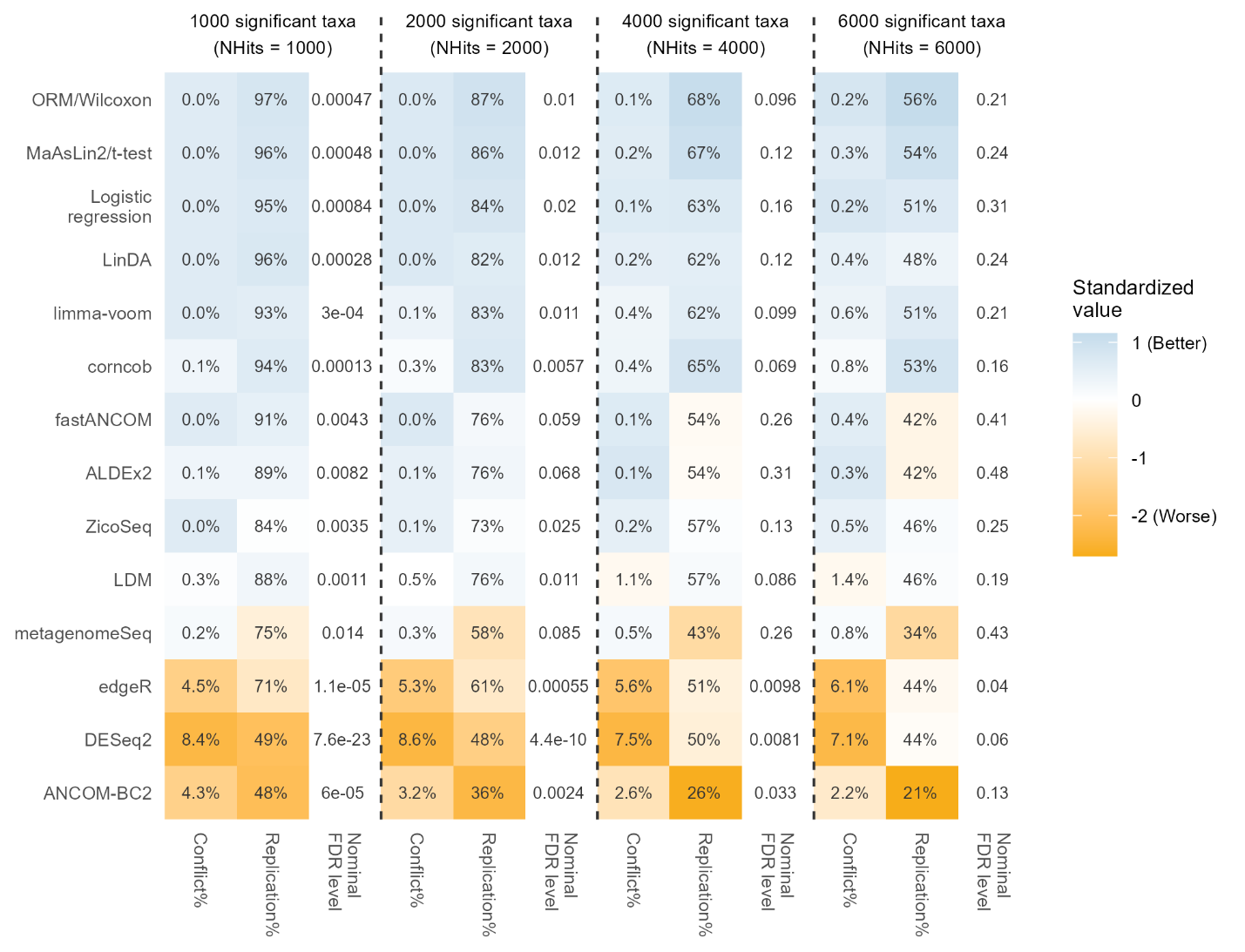
**

**Figure A3** The consistency of the 14 DAA methods when the nominal FDR levels were chosen so that each method detected a total of 1000, 2000, 4000 or 6000 significant taxa in the 285 exploratory datasets in the split-data analyses. The methods are in rank order based on the mean of the standardized values of the percentage of conflicting results (Conflict%) and replication percentage (Replication%) on all the four values of total number of significant taxa. (Conflict% was square root transformed before the standardization.) The number 6000 is approximately the number of significant taxa that the most sensitive methods (DESeq2 and edgeR) detected with nominal FDR level .05. (For ANCOM-BC2, the sensitivity filter was employed with FDR level .20. See Details on running the DAA methods.)

**
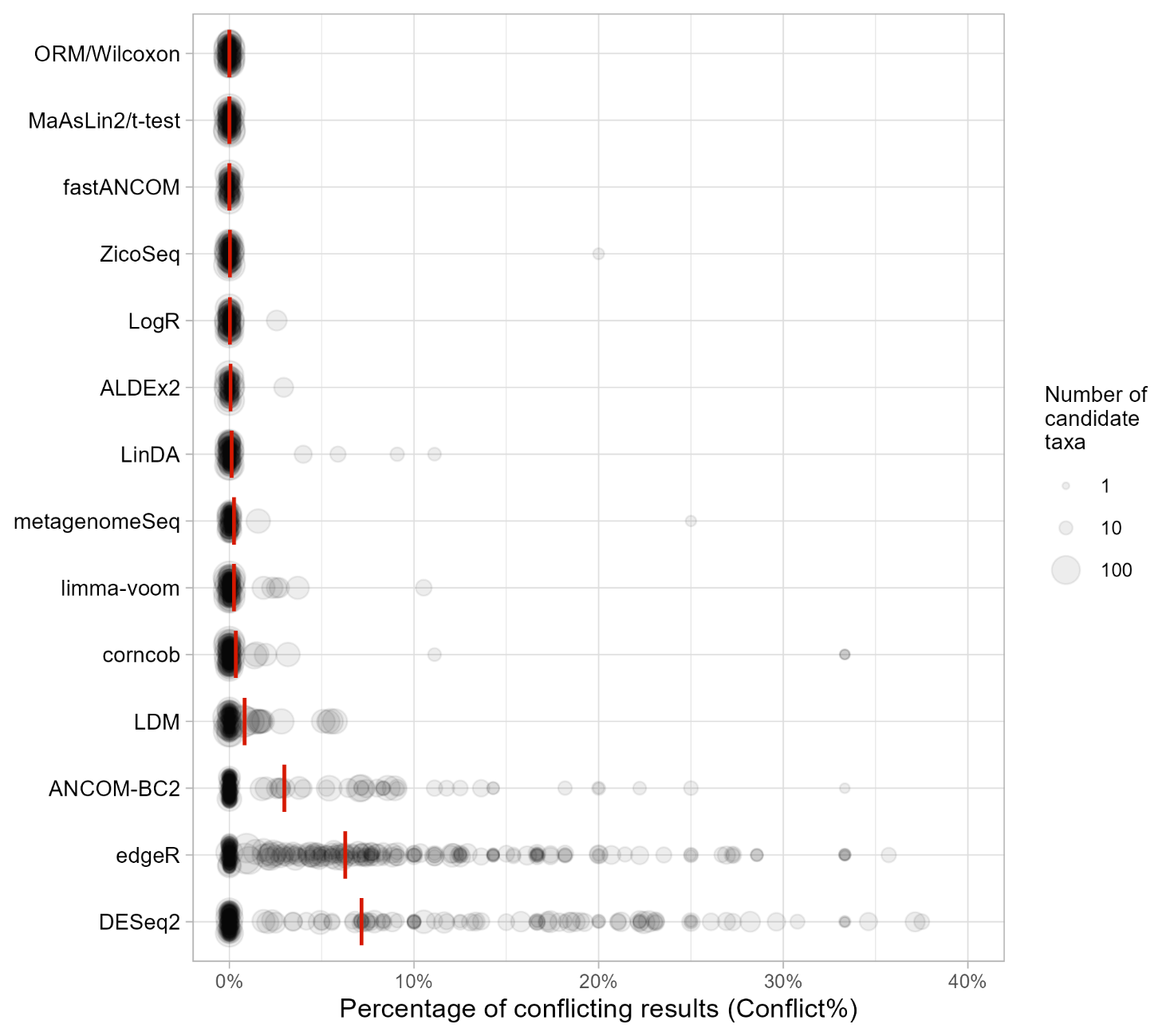
**

**Figure A4.1** The percentage of conflicting results (Conflict%) in the 285 pairs of datasets (with at least one candidate taxon) in the split-data analyses. The red lines indicate the overall Conflict%. The methods are ordered according to the overall values. The Figure is cut at 40%. Nominal FDR level = .05 was employed in the exploratory datasets.


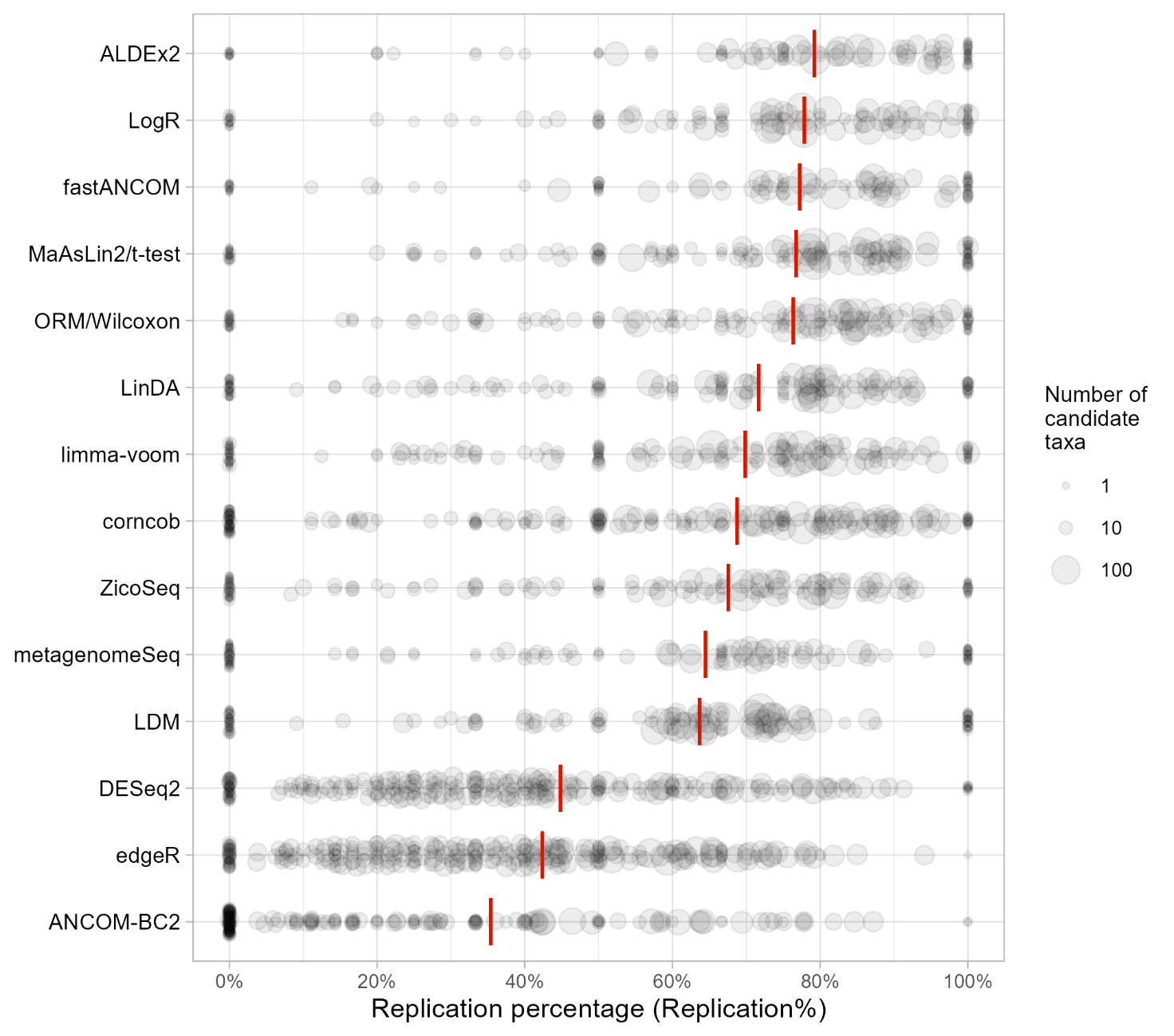


**Figure A4.2** The replication percentages (Replication%) in the 285 pairs of datasets (with at least one candidate taxon) in the split-data analyses. The red lines indicate the overall Replication%. The methods are ordered according to the overall values. Nominal FDR level = .05 was employed in the exploratory datasets.

**
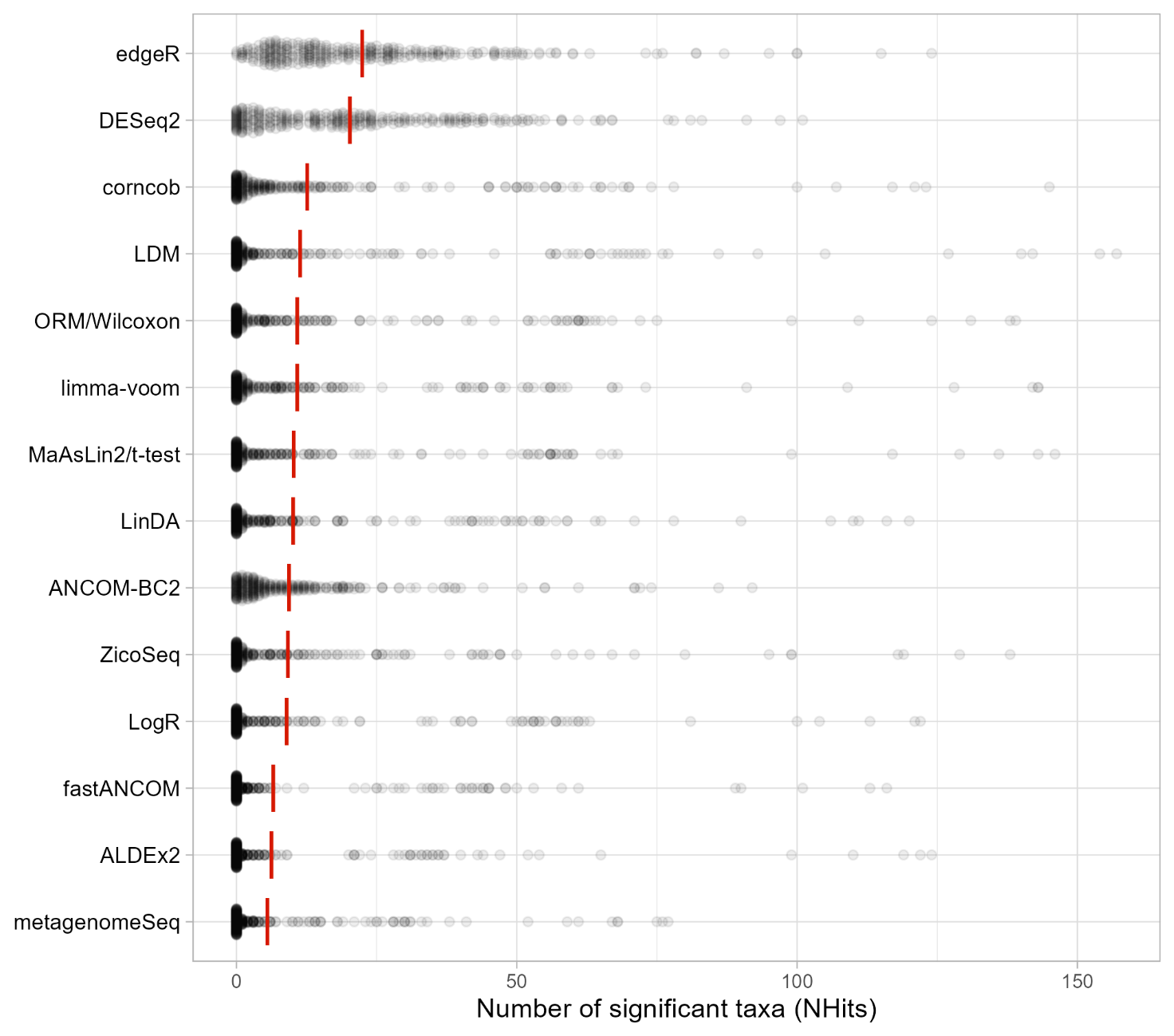
**

**Figure A4.3** The number of significant taxa (FDR adjusted p < .05) in the 285 exploratory datasets in the split-data analyses. The red lines indicate the mean numbers of significant taxa detected in the exploratory datasets. The methods are ordered according to these mean numbers.

**
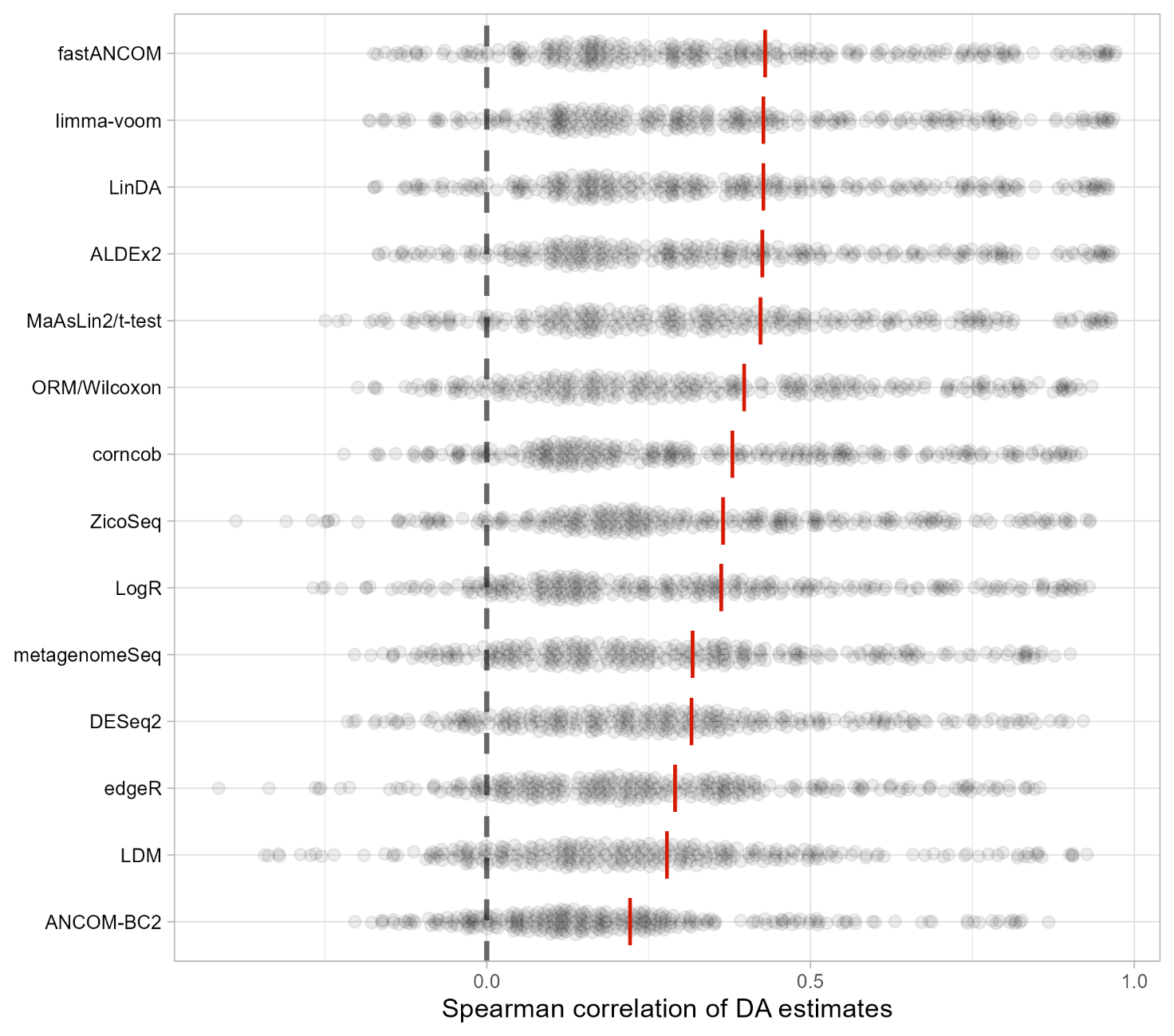
**

**Figure A5** The Spearman correlation coefficients of the DA estimates between exploratory and validation datasets in the split-data analyses. Values on all 285 exploratory-validation pairs of datasets are shown. The red lines indicate the average correlations, namely, the hyperbolic tangent transformed means of the inverse hyperbolic tangent transformed correlation coefficients. The methods are ordered according to the average correlation.

**
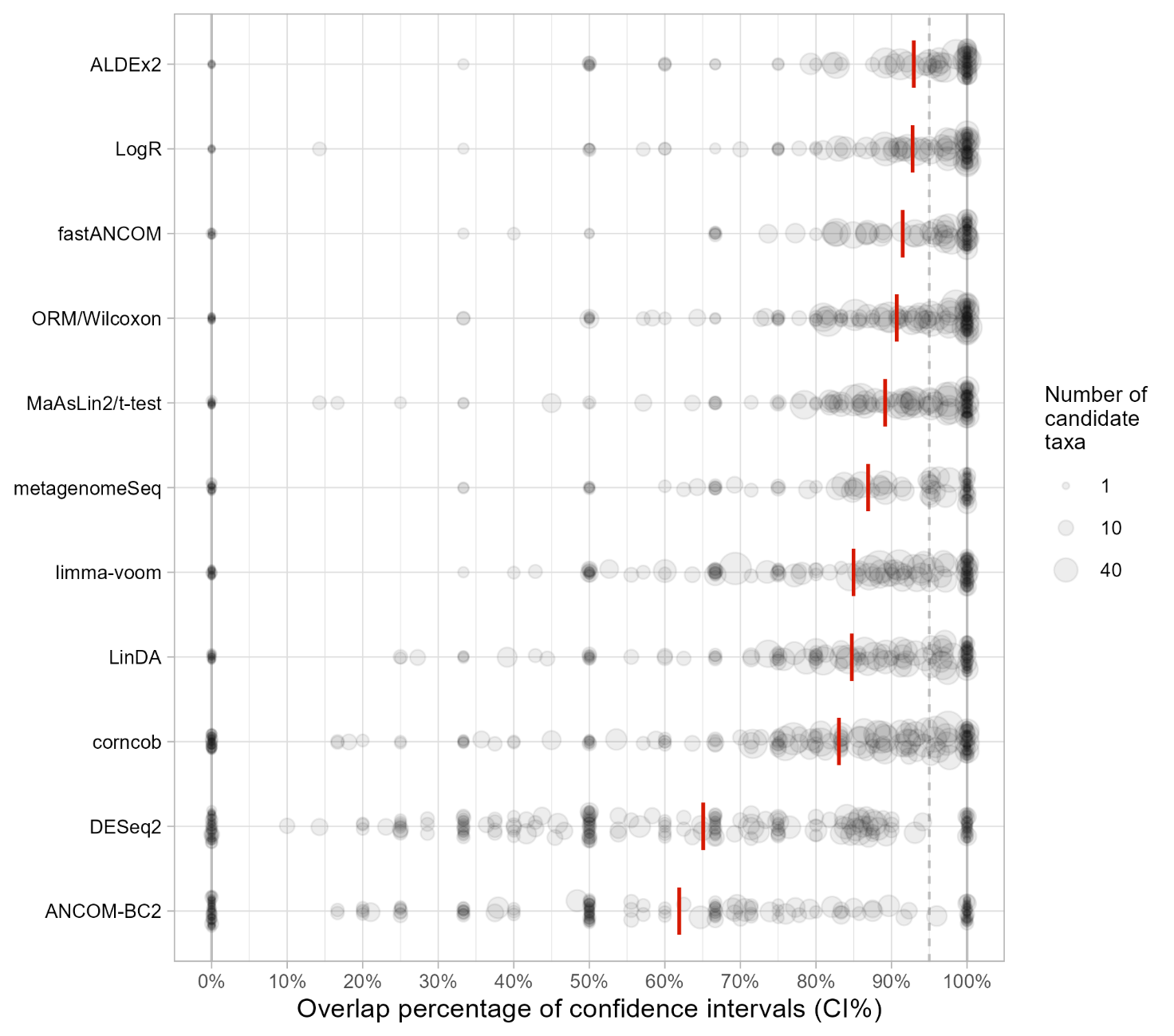
**

**Figure A6** The overlap percentage of 83.4% confidence intervals in the 285 pairs of datasets (with at least one candidate taxon) in the split-data analyses. Each grey point indicates the overlap percentage on a pair of datasets. The red lines indicate the overlap percentages (CI%) calculated over all candidate taxa in all pairs of datasets. The methods are ordered according to the overall value. The grey dashed line at 95% indicates a theoretical overlap percentage if DA estimates were normally distributed.

**
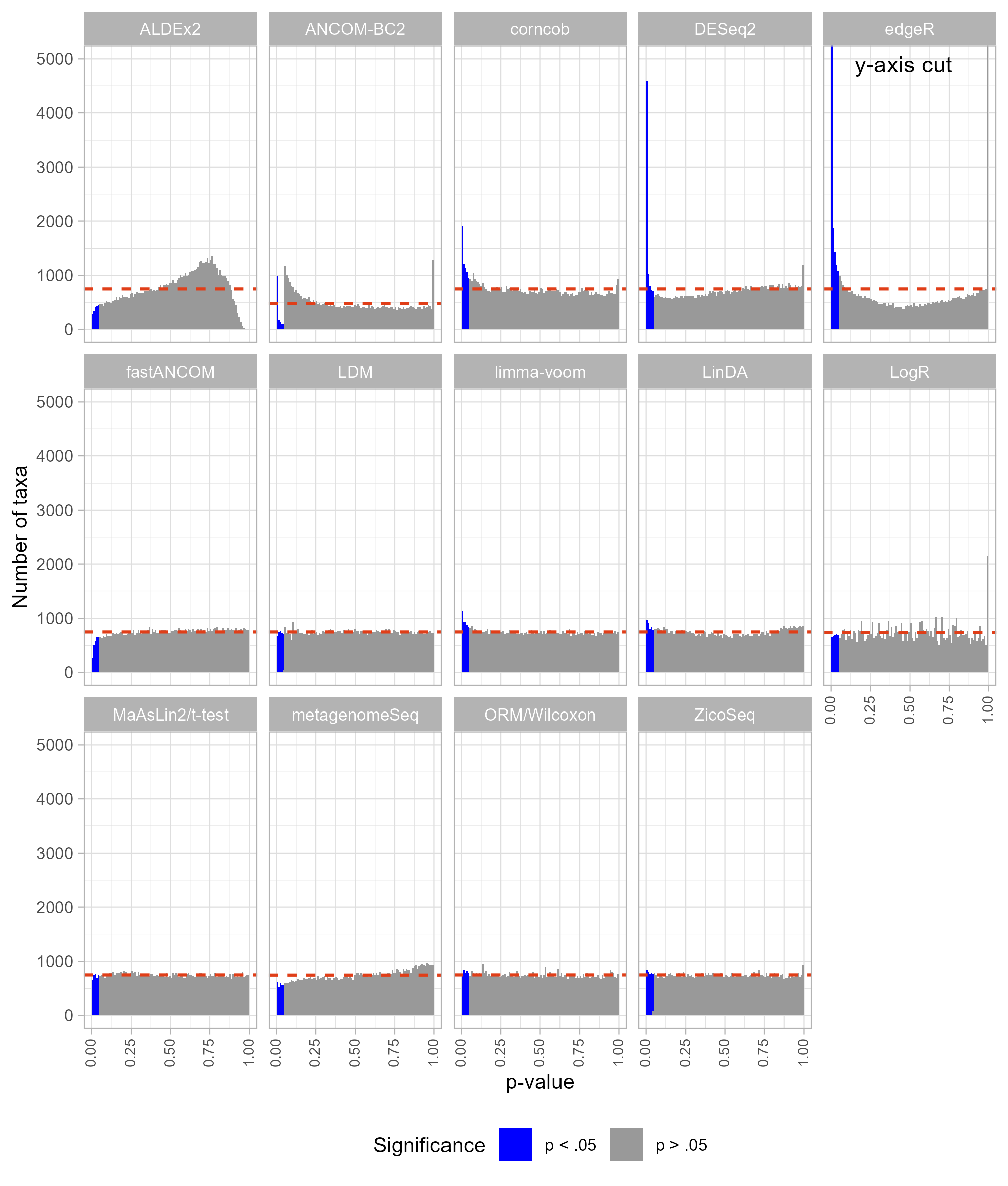
**

**Figure A7** The distribution of p-values (not adjusted for multiple testing) provided by the 14 DAA methods when run on 500 microbiome datasets with *randomly permuted* group labels (case/control). The width of each bin is .01. In this scenario, where there are no truly differentially abundant taxa, an ideally performing method should provide uniformly distributed p-values (indicated by the dashed red lines). Especially, the number of p-values < .05 (in blue color) should be at (or below) the red dashed line. For visualization purposes the y-axis is cut at 5000 taxa. For edgeR, the number of taxa with p < .05 is 9241. For ANCOM-BC2, the p-values that do not pass the sensitivity analysis for zeros (at p < .05) are filtered out. The 500 datasets were constructed by randomly permuting the group labels 10 times on the 50 real datasets included in the separate study analyses. The percentage of p < .05 for each method: ALDEx2 (2.5%), ANCOM-BC2 (3.1%), corncob (8.4%), DESeq2 (10.5%), edgeR (18.4%), fastANCOM (3.6%), LDM (4.8%), limma-voom (6.3%), LinDA (5.8%), LogR (4.6%), MaAsLin2/t-test (4.8%), metagenomeSeq (3.9%), ORM/Wilcoxon (5.3%), ZicoSeq (5.2%).

**
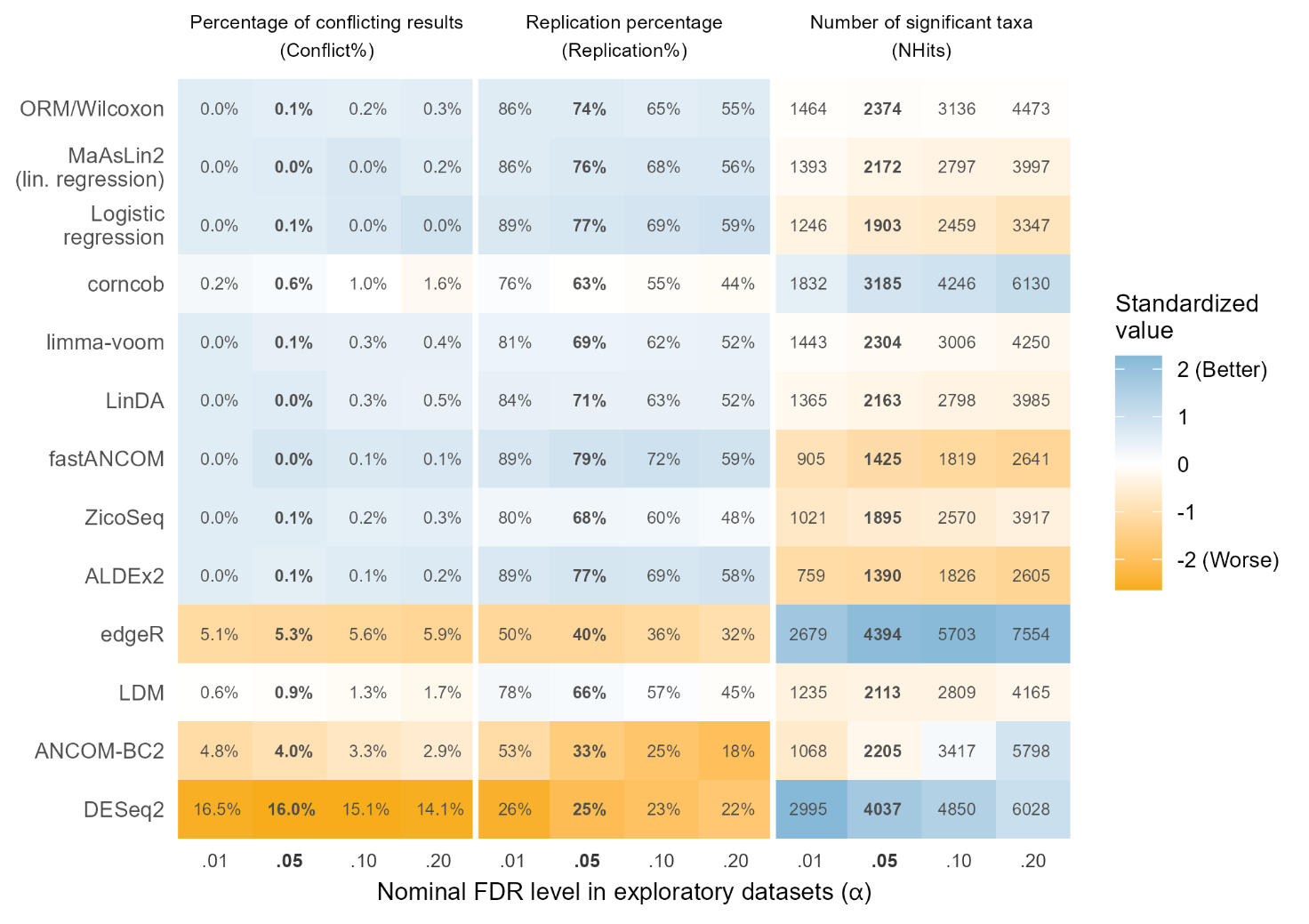
**

**Figure A8** The results of the split-data analyses when covariates (age, sex or BMI) are included in DAA. The methods are in rank order based on the mean of the standardized values of the metrics. Values based on the nominal FDR level α = .05 are shown in bold. Altogether 45 original datasets for which at least one of the covariates age, sex or BMI was available were used in the analysis. Each original dataset was split five times to form pairs consisting of an exploratory and a validation dataset, thus totaling 225 (= 45 × 5) pairs of datasets. Candidate taxon = a taxon that was significant (FDR adjusted p < α) in an exploratory dataset and present in the validation dataset. Conflict% = the percentage of candidate taxa that were significant (p < .05) in the validation dataset, but in the opposite direction to that in the exploratory dataset. Values below 0.025%, 0.125%, 0.25% and 0.50% were considered ideal for α = .01, .05, .10 and .20, respectively. Replication% = the percentage of candidate taxa that were significant (p < .05) in the validation dataset in the same direction as in the exploratory dataset. NHits = the total number of significant taxa found in the 225 (45 × 5) exploratory datasets. A higher value of NHits can be considered better when it is accompanied by a low Conflict% and a high Replication%.

**
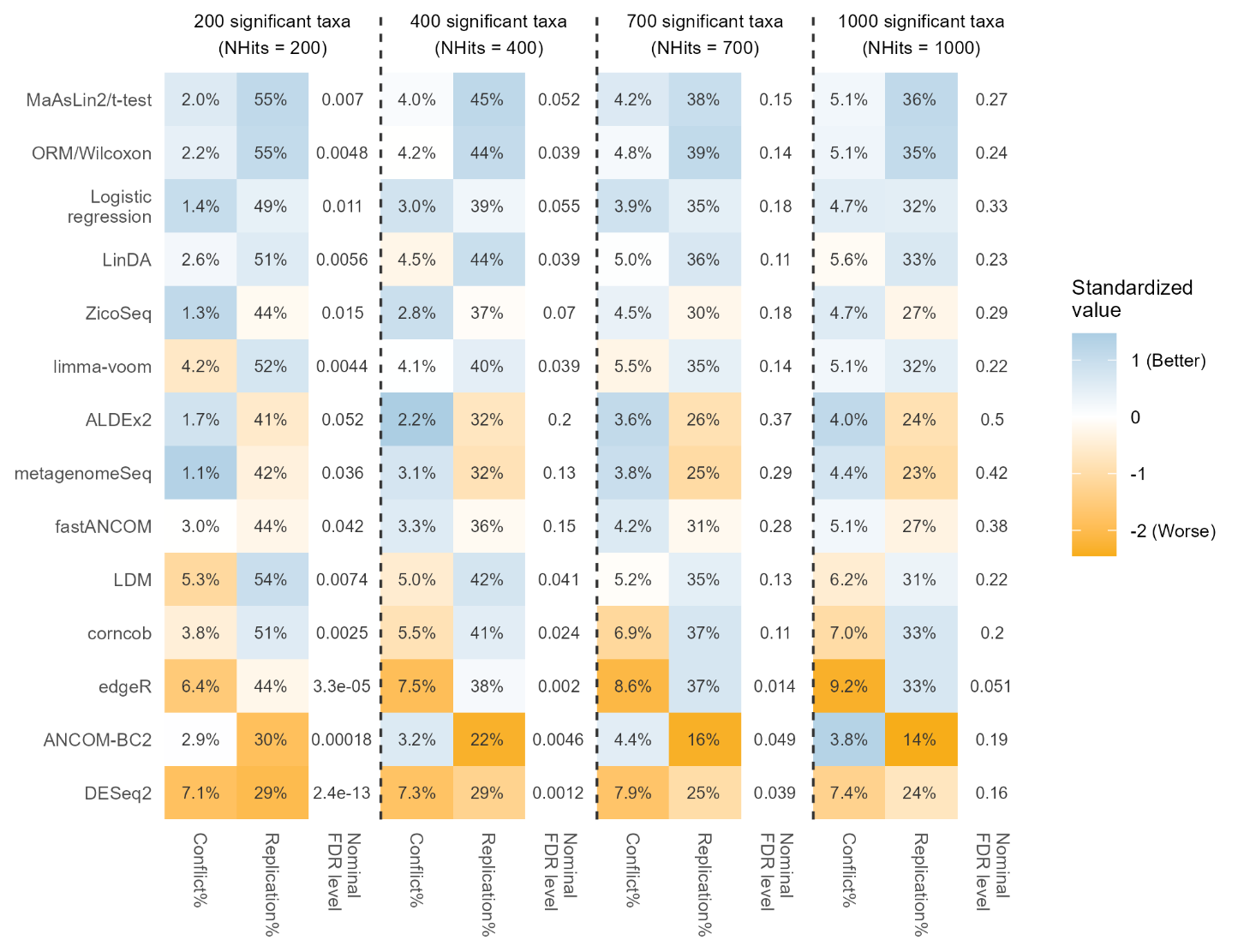
**

**Figure A9** The consistency of the 14 DAA methods when the nominal FDR levels were chosen so that each method detected a total of 200, 400, 700 or 1000 significant taxa in the 37 exploratory datasets in the separate study analyses. The methods are in rank order based on the mean of the standardized values of the percentage of conflicting results (Conflict%) and replication percentage (Replication%) on all the four values of total number of significant taxa. (Conflict% was square root transformed before the standardization.) The number 1000 is approximately the number of significant taxa that the most sensitive method (edgeR) detected with nominal FDR level .05. (For ANCOM-BC2, the sensitivity filter was employed with FDR level .20. See Details on running the DAA methods.)


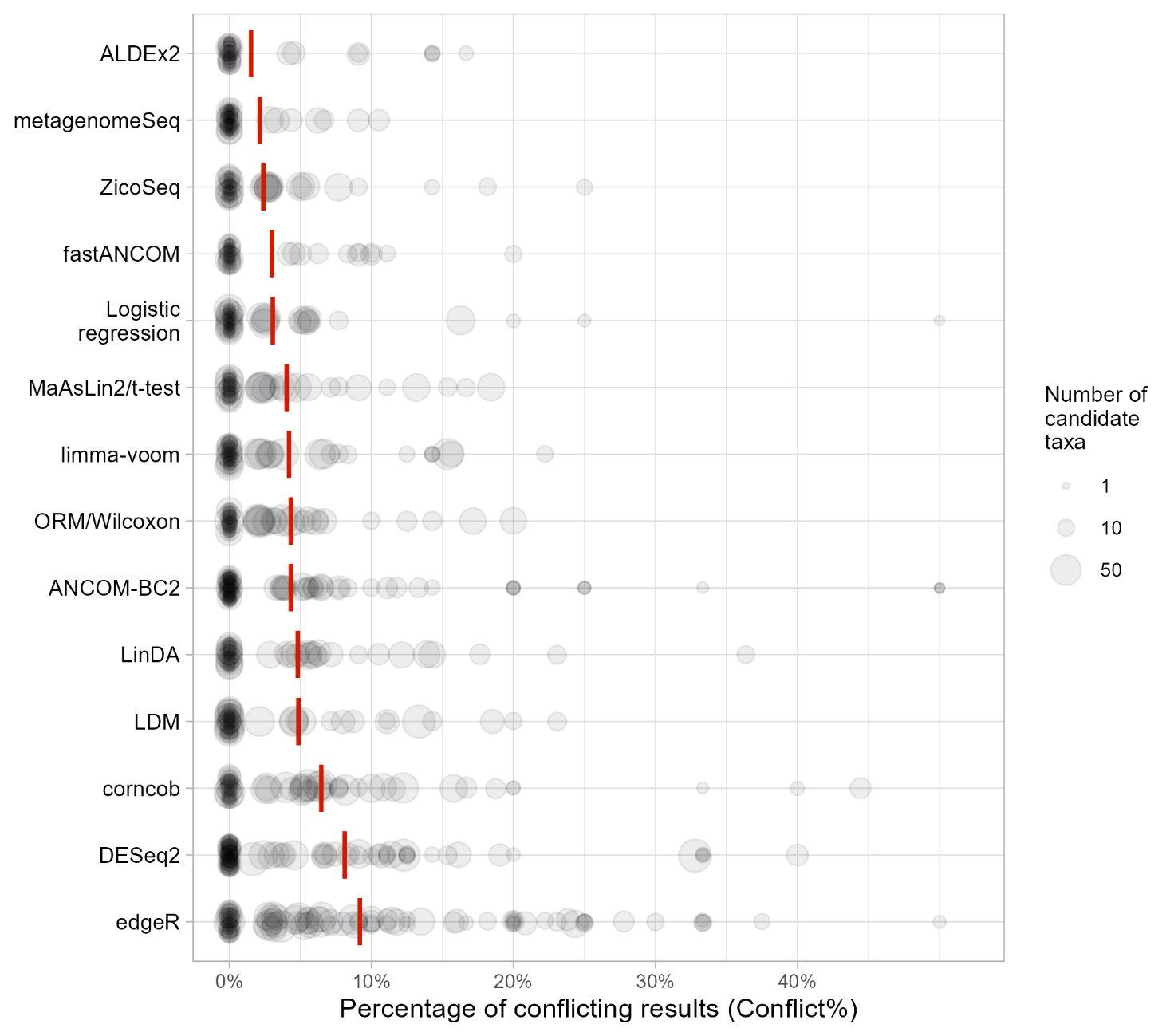


**Figure A10.1** The percentage of conflicting results (Conflict%) in all pairs of datasets (with at least one candidate taxon) in the separate study analyses. The red lines indicate the overall Conflict%. The methods are ordered according to the overall values. The figure is cut at 52%. Nominal FDR level = .05 was employed in the exploratory datasets.


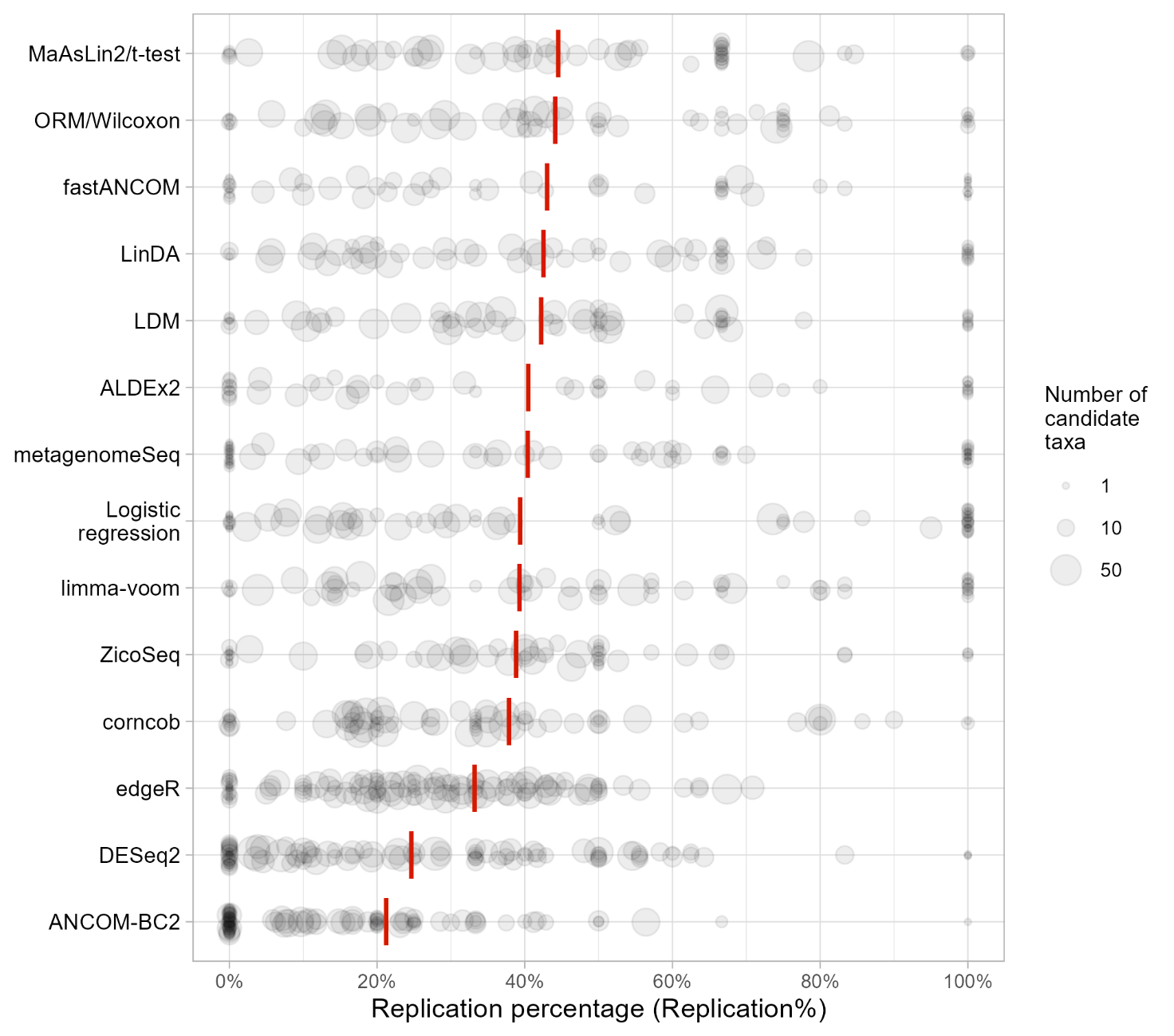


**Figure A10.2** The replication percentages (Replication%) in all pairs of datasets (with at least one candidate taxon) in the separate study analyses. The red lines indicate the overall Replication%. The methods are ordered according to the overall values. Nominal FDR level = .05 was employed in the exploratory datasets.

**
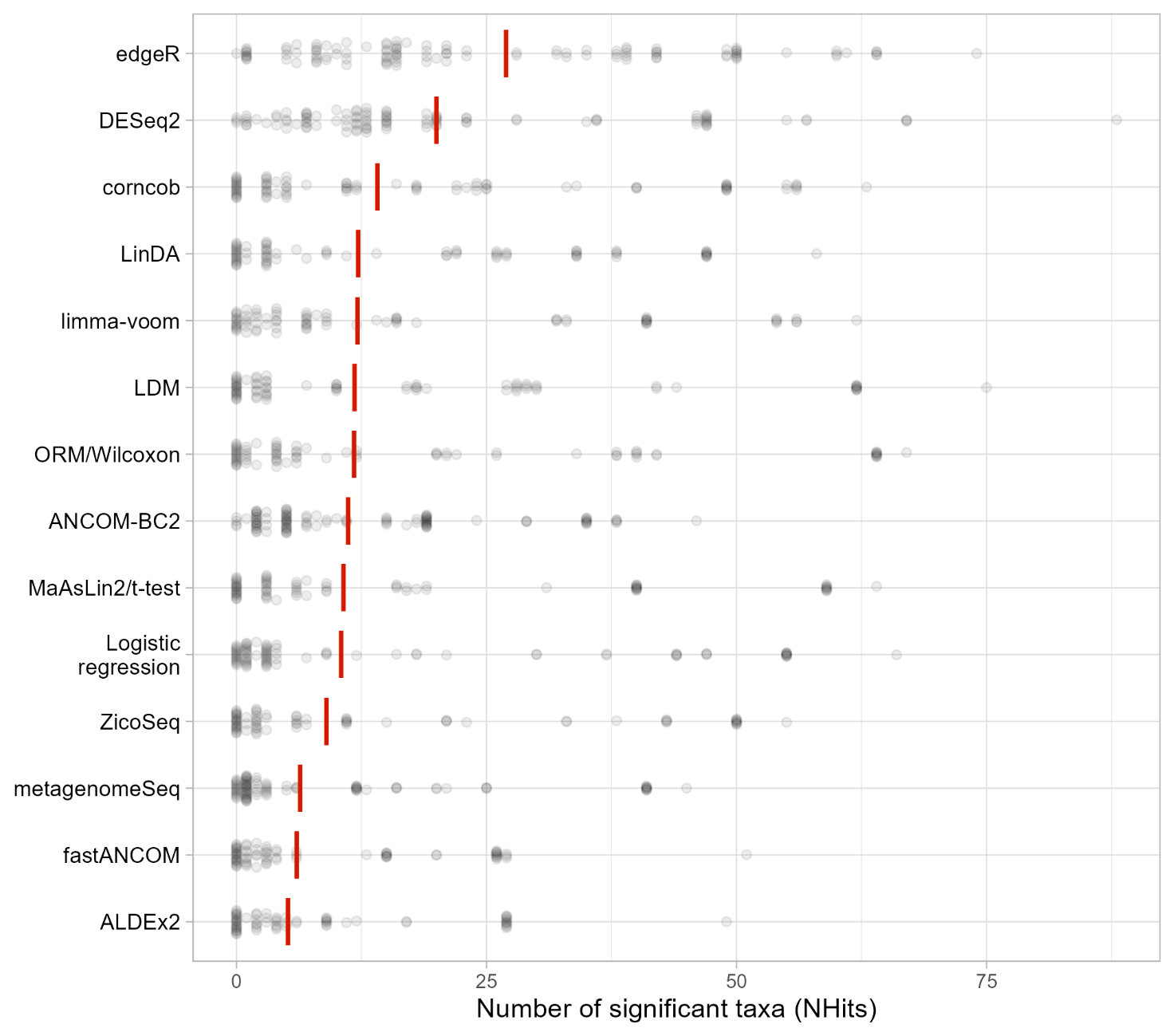
**

**Figure A10.3** The number of significant taxa (FDR adjusted p < .05) in the 37 exploratory datasets in the separate study analyses. The red lines indicate the mean number of significant taxa detected in the exploratory datasets. The methods are ordered according to these mean numbers.

**
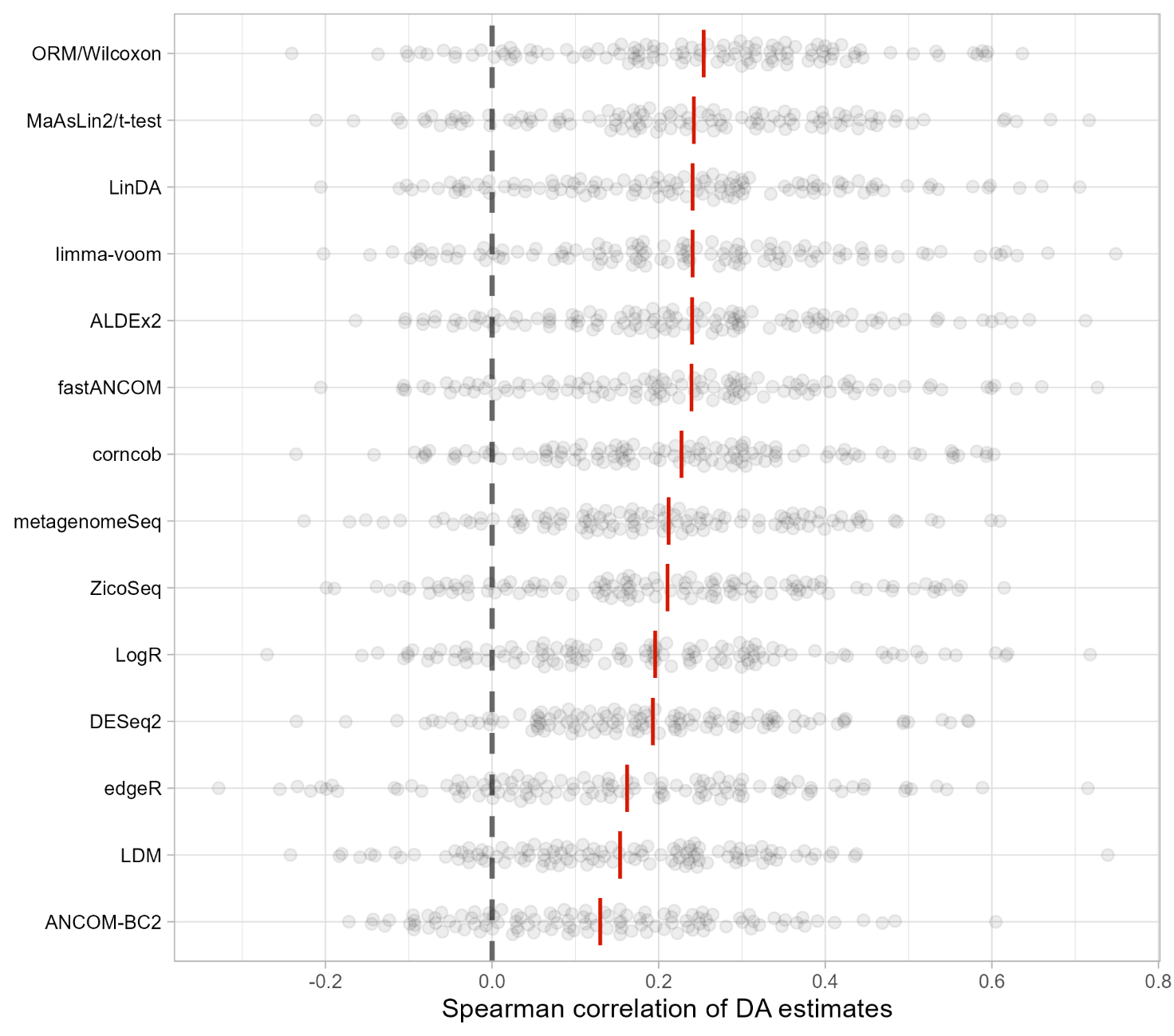
**

**Figure A11** The Spearman correlation coefficients of the DA estimates between the exploratory and validation datasets in the separate study analyses. The values in all exploratory-validation pairs of datasets are shown. The red lines indicate the average correlations, i.e., the hyperbolic tangent transformed mean of the inverse hyperbolic tangent transformed correlation coefficients. The methods are ordered according to the average correlation.

**
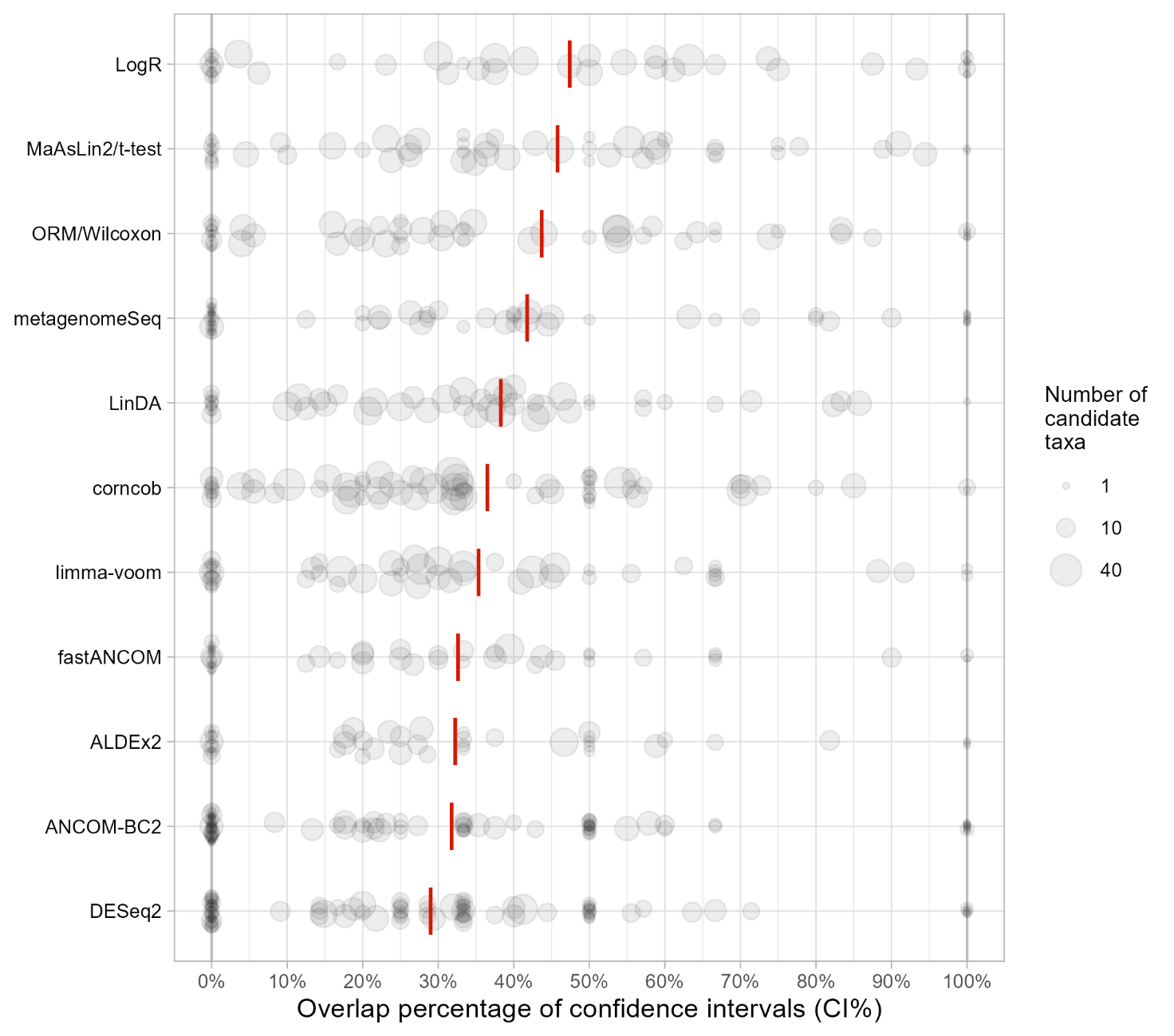
**

**Figure A12** The overlap percentage of 83.4% confidence intervals in all pairs of datasets (with at least one candidate taxon) in the separate study analyses. Each grey point indicates the overlap percentage in a pair of datasets. The red lines indicate the overall overlap percentages (CI%) calculated over all candidate taxa in all pairs of datasets. The methods are ordered according to the overall value.

**
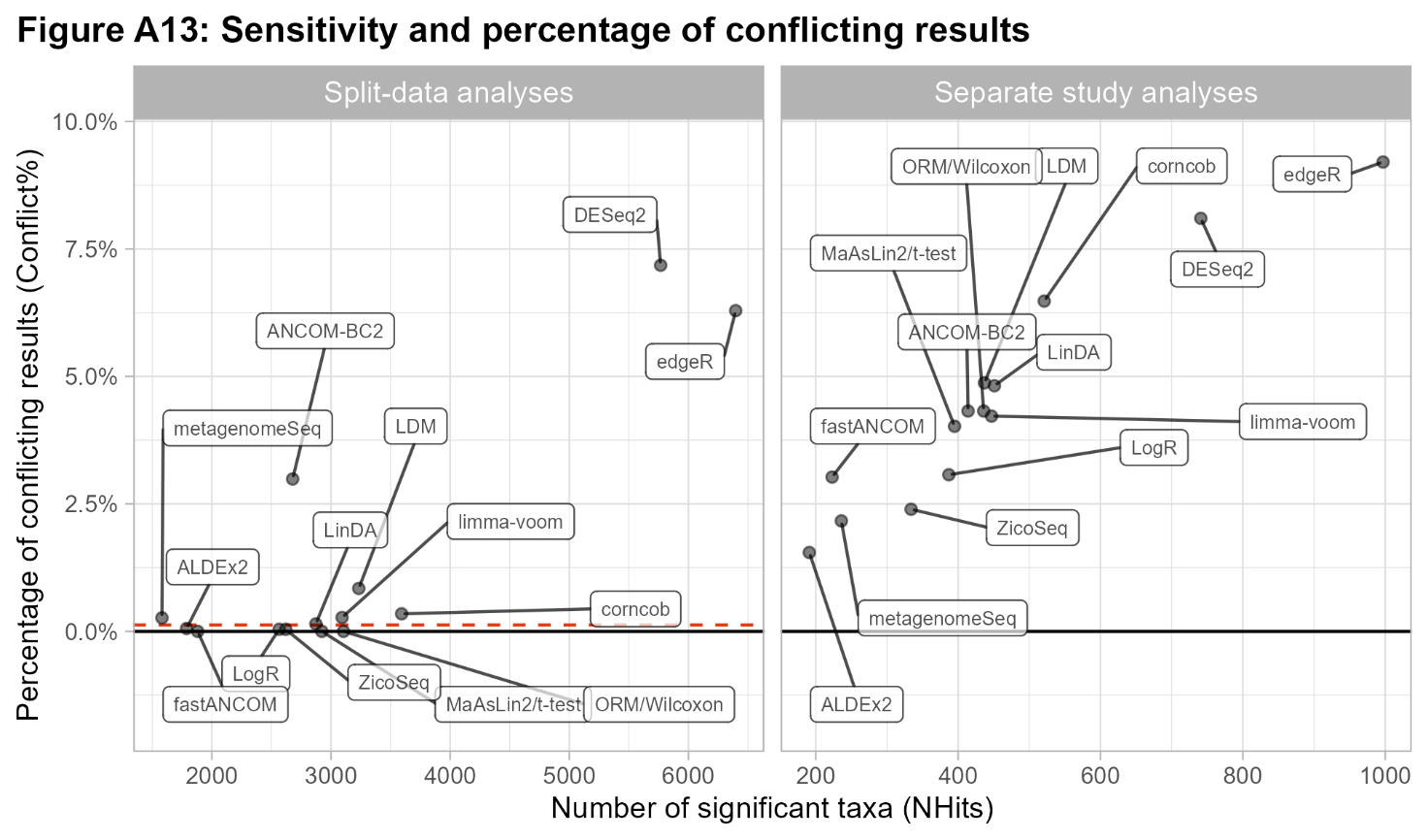
**

**Figure A13** This figure illustrates the correlation between the sensitivity and inconsistency of DAA methods. The sensitivity is measured by the total number of significant taxa found in all exploratory datasets (NHits). The inconsistency is measured by the percentage of conflicting results (Conflict%). Nominal FDR level α = .05 is used in both subfigures. The red dashed line on the left indicates the level of acceptable Conflict% (.125%).

**Results for additional methods and alternative versions of the 14 DAA methods**

The figures A14.1 – A14.4 below correspond Figures 2 - 5 in the main text. For details on the shown methods see the subsection “Details on running the DAA methods” above. The additional methods or versions are

- ALDEx2 with scale uncertainty (ALDEx2-scale) [51]
- ALDEx2 based on Wilcoxon test (ALDEx2-Wilcox)
- corncob with unequal variances allowed (corncob-UEV)
- DESeq2 with p values based on likelihood ratio test (DESeq2-LRT)
- LDM with CLR normalization (LDM-CLR)
- MaAsLin2 with arcsine square root transformation (MaAsLin2-AST)
- MaAsLin2 with CLR normalization (MaAsLin2-CLR)
- MaAsLin2 with CSS normalization (MaAsLin2-CSS)
- MaAsLin2 with TMM normalization (MaAsLin2-TMM)
- Negative binomial regression with TSS normalization (NegBin)
- ORM/Wilcoxon with GMPR normalization (ORM/W-GMPR)
- ORM/Wilcoxon with Wrench normalization (ORM/W-Wrench)
- radEmu [55]


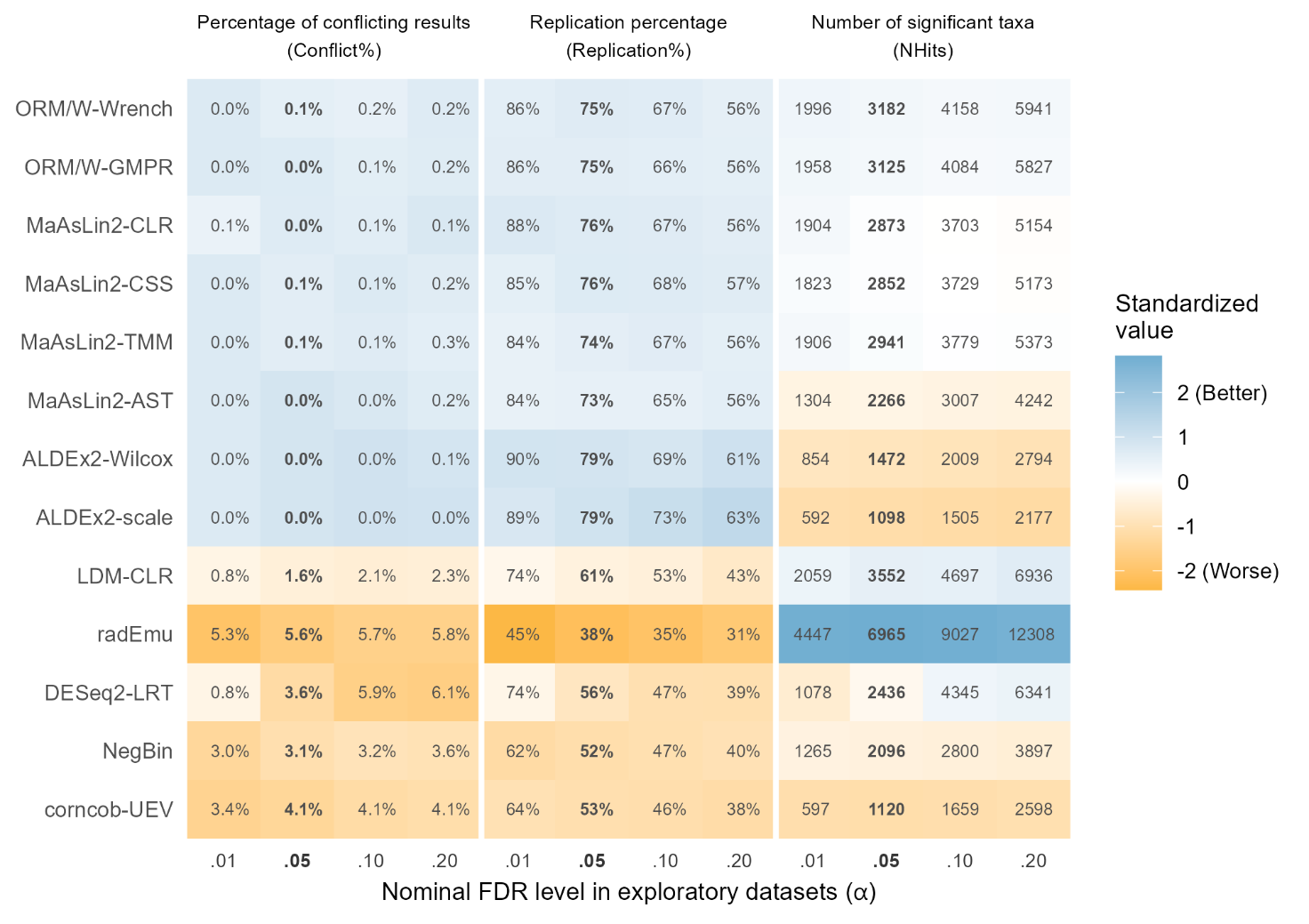


**Figure A14.1** The results for alternative versions of the methods in the split-data analyses. The figure corresponds to Figure 2 in the main text.


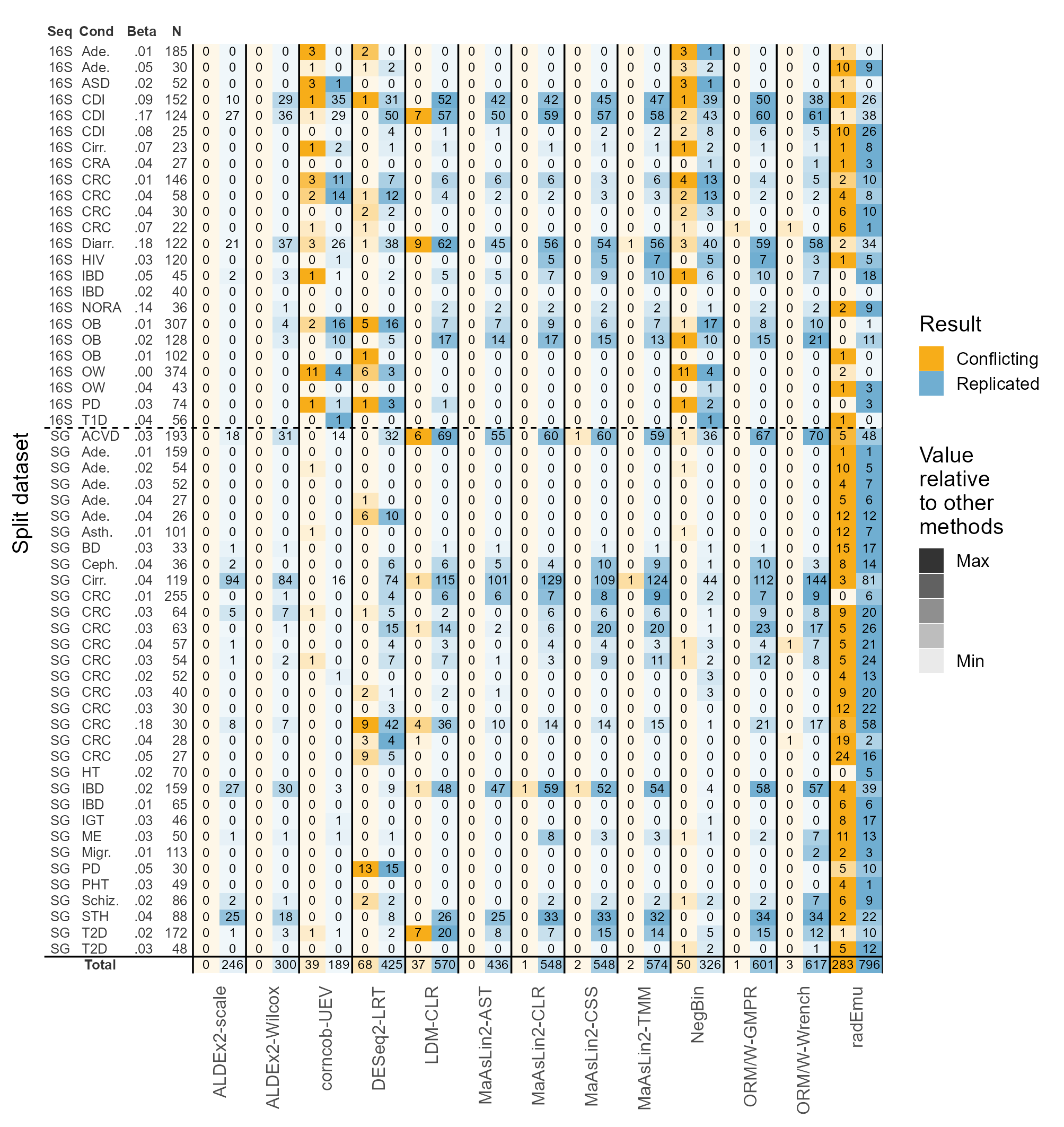


**Figure A14.2** The number of conflicting and replicated results in the split-data analyses for alternative versions of the methods. This figure corresponds to Figure 3 in the main text.


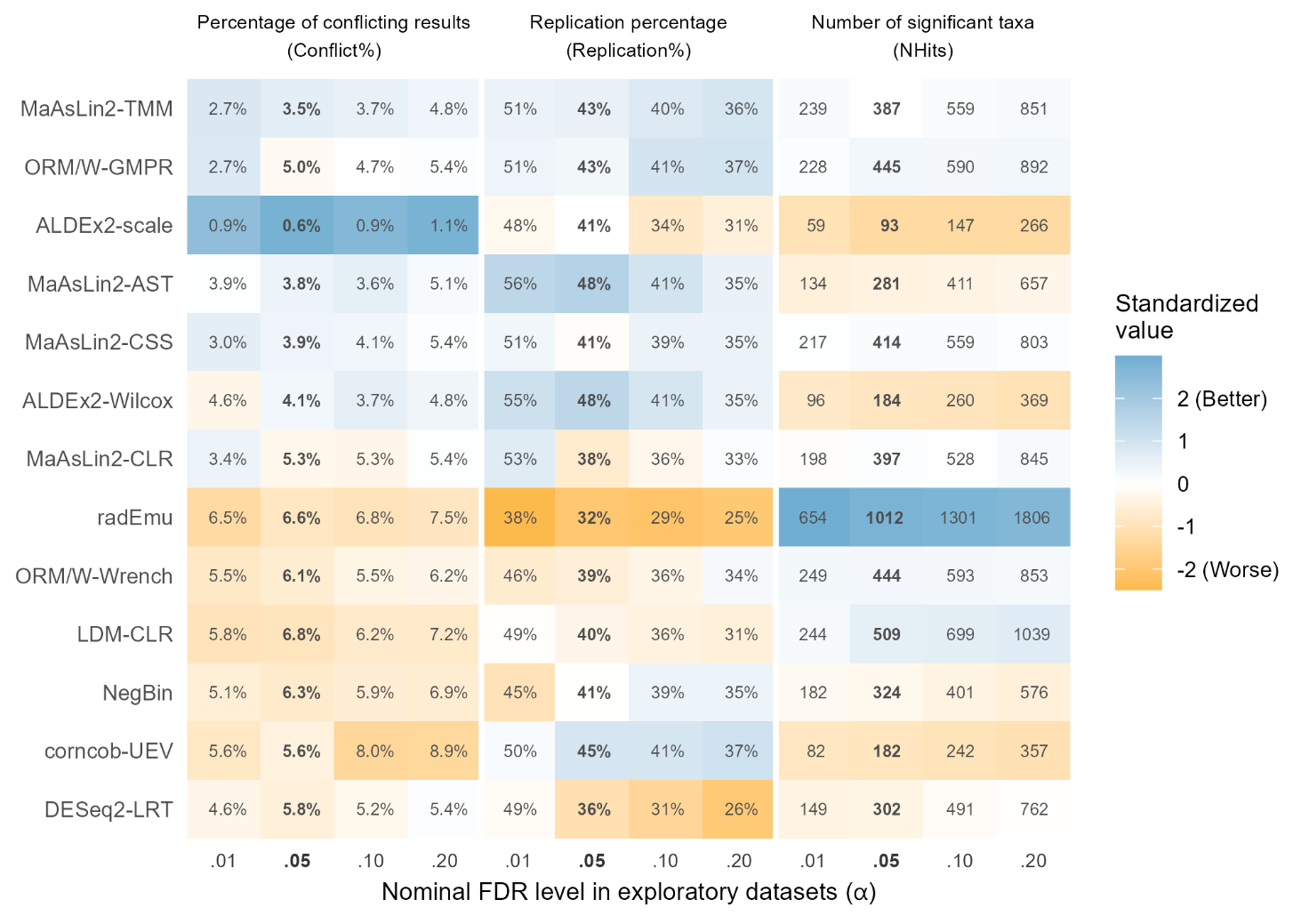


**Figure A14.3** The results for alternative versions of the methods in the separate study analyses. The figure corresponds to Figure 4 in the main text.


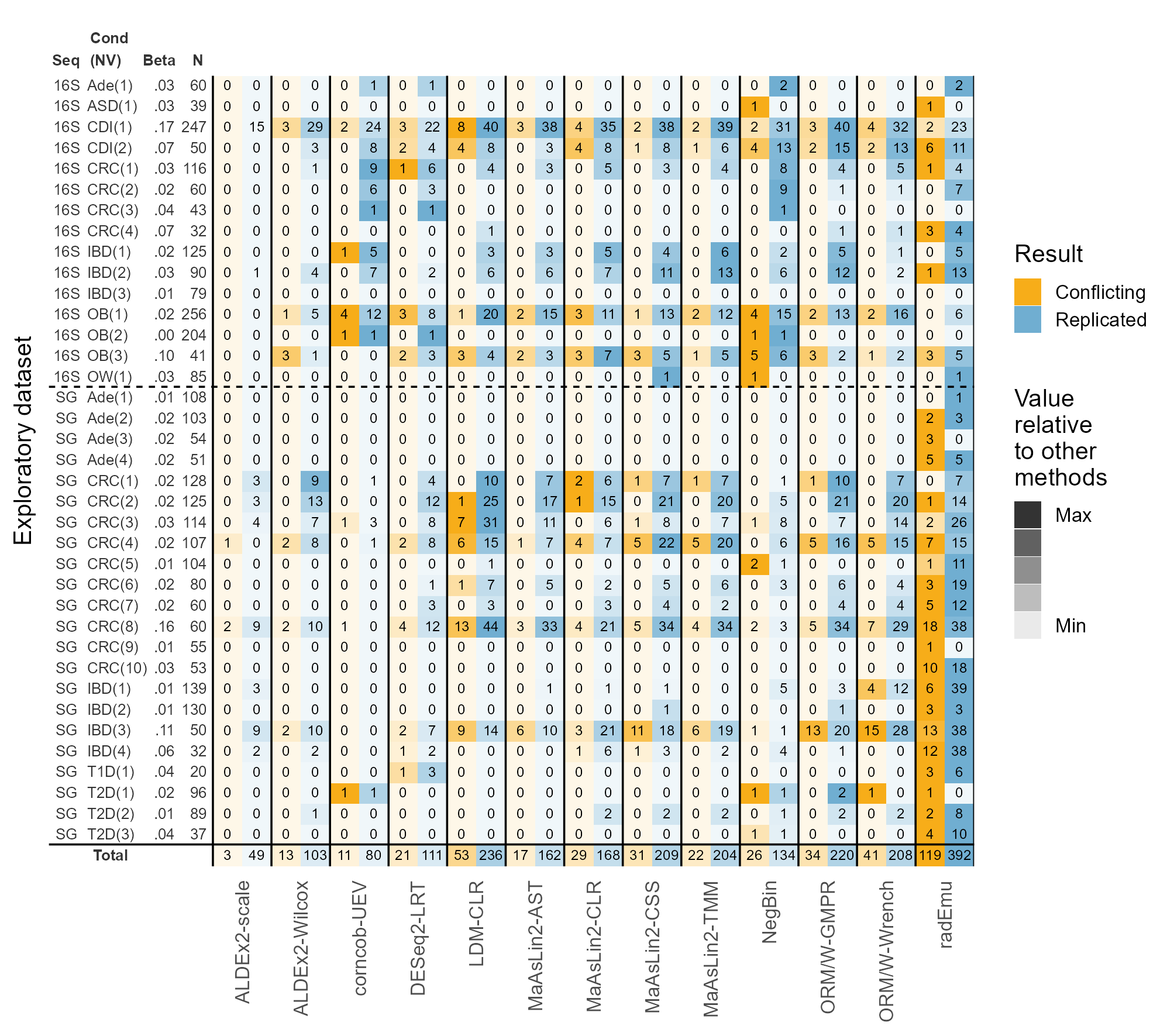


**Figure A14.4** The number of conflicting and replicated results in the separate study analyses for alternative versions of the methods. The figure corresponds to Figure 5 in the main text.

**Additional analysis to evaluate how different normalization strategies perform under** **large systematic differences in the total absolute abundances**

We here describe the additional analysis of how different methods can address the case of large systematic differences in total absolute abundances in practice. We used a real dataset from a gut microbiome study on 12 (6 + 6) mice measured at four time points [58]. In this study the absolute microbial abundances were measured to be clearly systematically higher in one group. We performed DAA with each method on this dataset in the standard way using only the observed counts. We then compared the direction of DAA estimates provided by the methods to the “true” directions. For each taxon, the “true” direction was defined as the sign of the difference of the arithmetic means of the measured absolute abundances. If the mean absolute abundance was greater in the case group, the sign was positive (otherwise it was negative). For each method, we then calculated the accuracy of estimating the sign correctly (accuracy = correct signs / number of taxa).

The results are shown in Figure A15. The methods employing TSS normalization perform generally well. Furthermore, methods employing CLR-transformation *in some phase* of DAA perform generally below average.


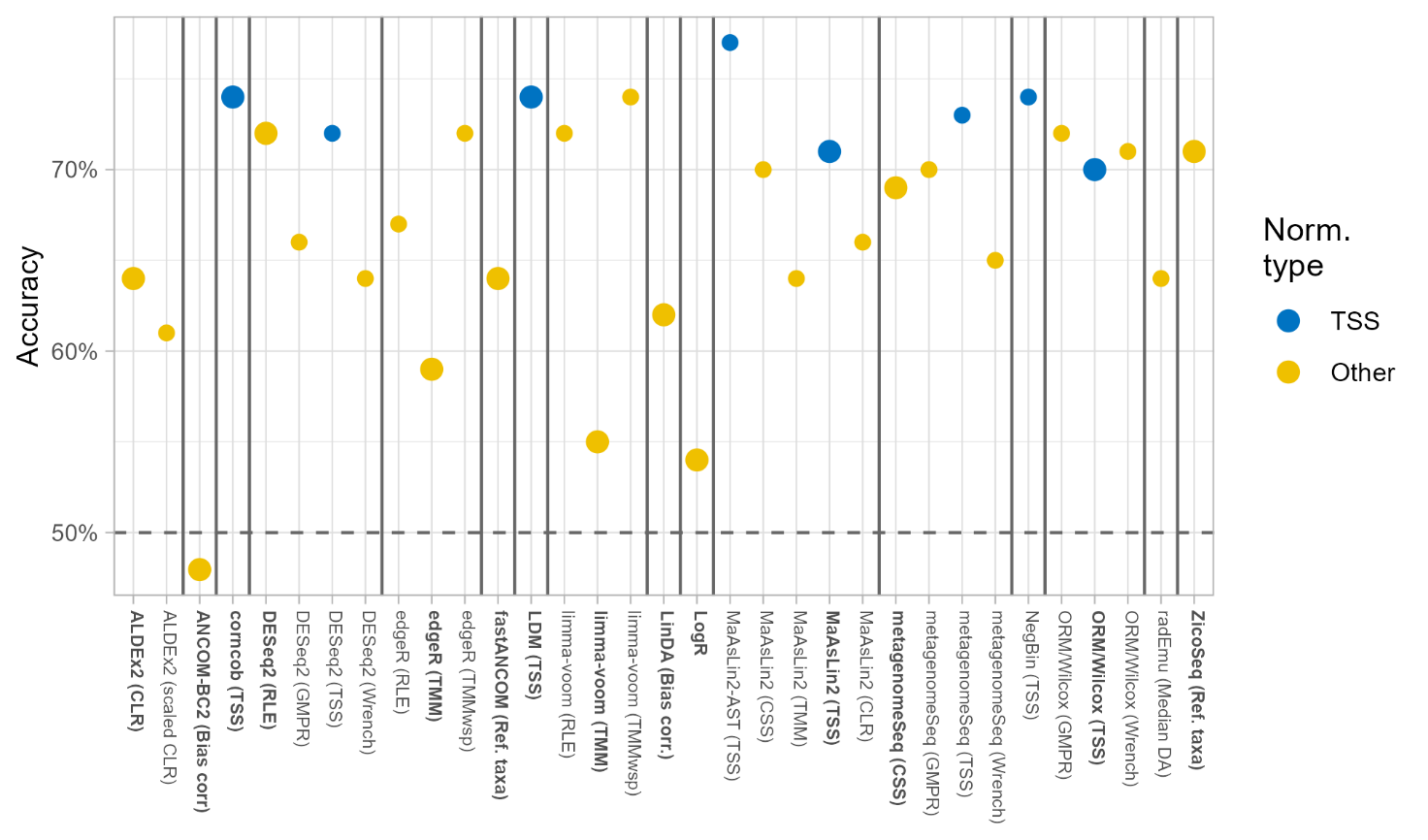


**Figure A15** The figure illustrates the ability of DAA methods to estimate the direction of “true” DA based on measured absolute abundances on a real dataset from a microbiome study on mice. The bolded names and larger points indicate the versions of the methods that are included in the main text. The normalization method/strategy is given in parenthesis. Norm. type indicates whether TSS normalization (or its equivalent) was used or whether some other type of normalization strategy was employed.

**Additional analyses to evaluate how different normalization strategies affect the performance of DAA methods**

Here we simply evaluated the methods based on the statistical significance of the results (q < .05) they provided on 50 datasets used in the separate study analyses. Jaccard distances based on significance of the results were calculated and Multidimensional Scaling (MDS) analysis based on those distances was performed. The results for the two most important MDS coordinates are shown below (Figure A16a). We only show results for the more appropriately performing methods (e.g. DESeq and edgeR would show rather far away from other methods). In Figure A16b we show the results only for datasets from studies investigating CDI (Clostridium difficile infection) as we consider it likely that there may occur systematic differences in the total absolute abundances.

The results show that, generally, other factors than normalization method (apart from CLR normalization) affect mostly the findings made by a method, especially for MaAsLin2 and ORM/Wilcoxon.


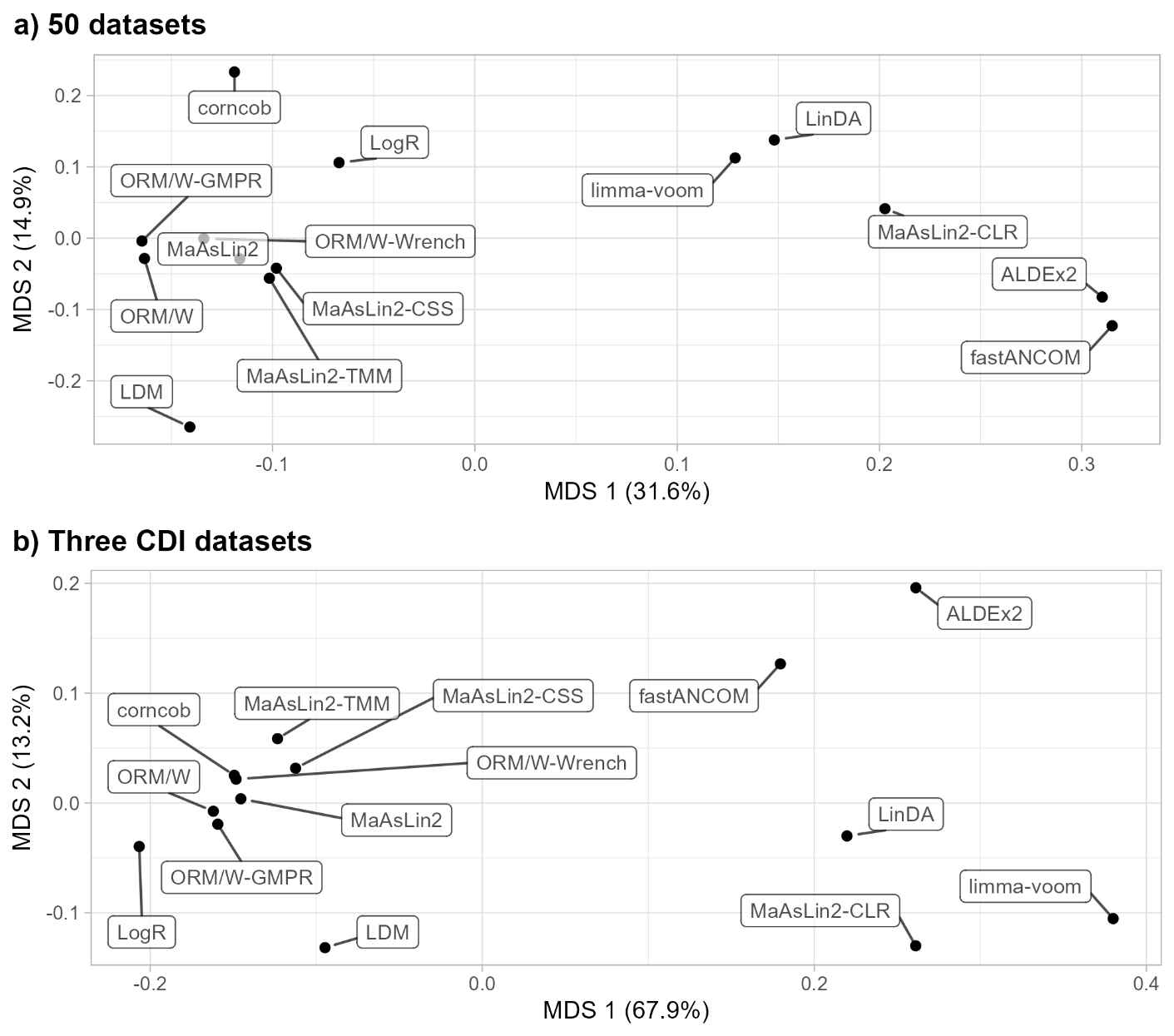


**Figure A16** DAA methods clustered based on Jaccard distances which are calculated based on statistical significance of the results provided by the methods on 50 datasets (a) and on three 3 16S datasets from studies investigating CDI (Clostridioides difficile infection) (b). The variance explained by the principal coordinate is shown in the parentheses. ORM/W = ORM/Wilcoxon. If some non-default normalization method was employed, it is shown after the name of the method.


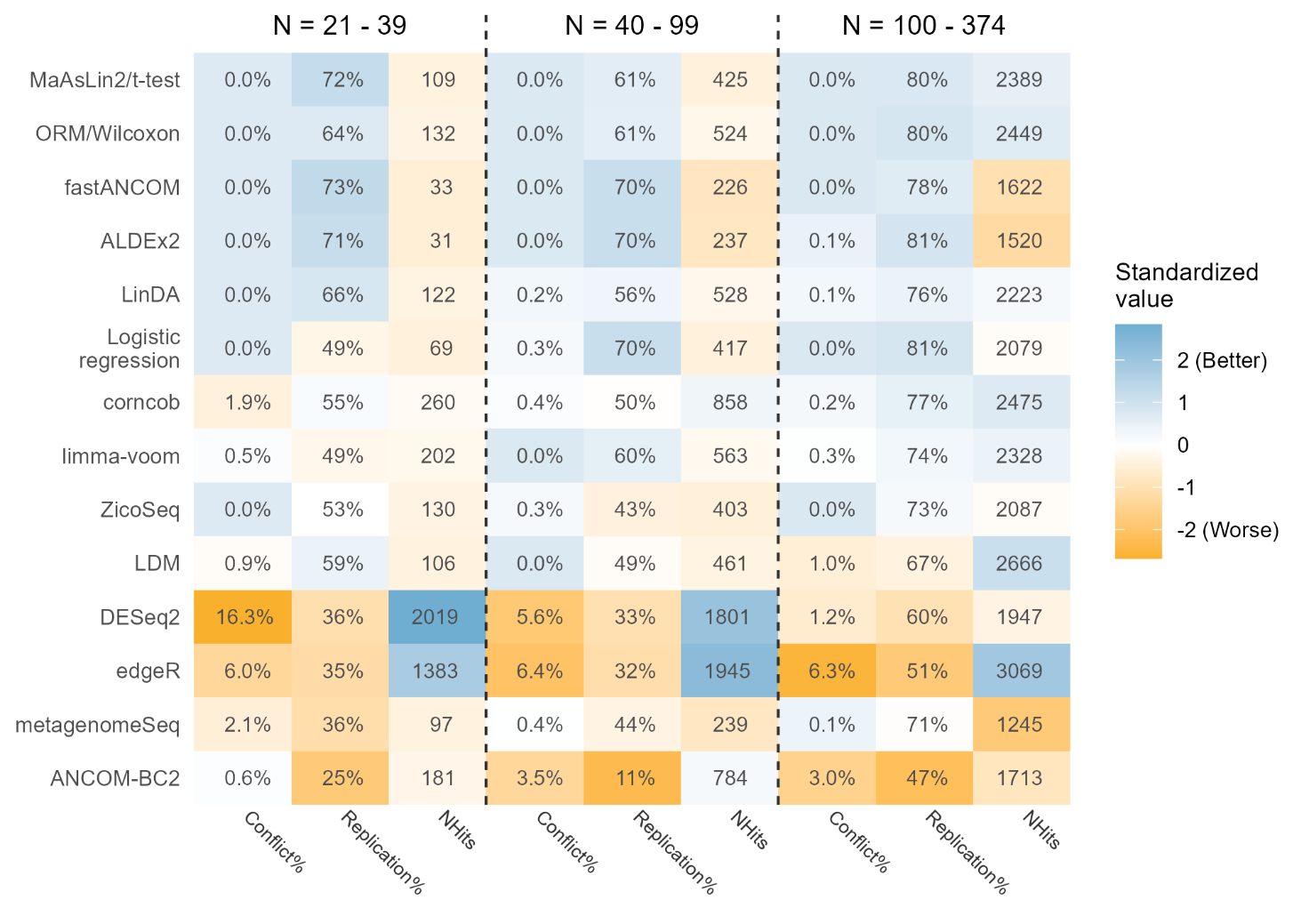


**Figure A17.1** The main results of the split-data analyses, stratified by sample size (N). The sample size refers to the number of subjects in a single exploratory or validation dataset. There were 5 x 18, 5 x 22 and 5 x 17 exploratory and validation datasets with sample sizes between 21 and 39, between 40 and 99, and between 100 and 374, respectively. FDR level α = .05 was employed in the exploratory datasets.


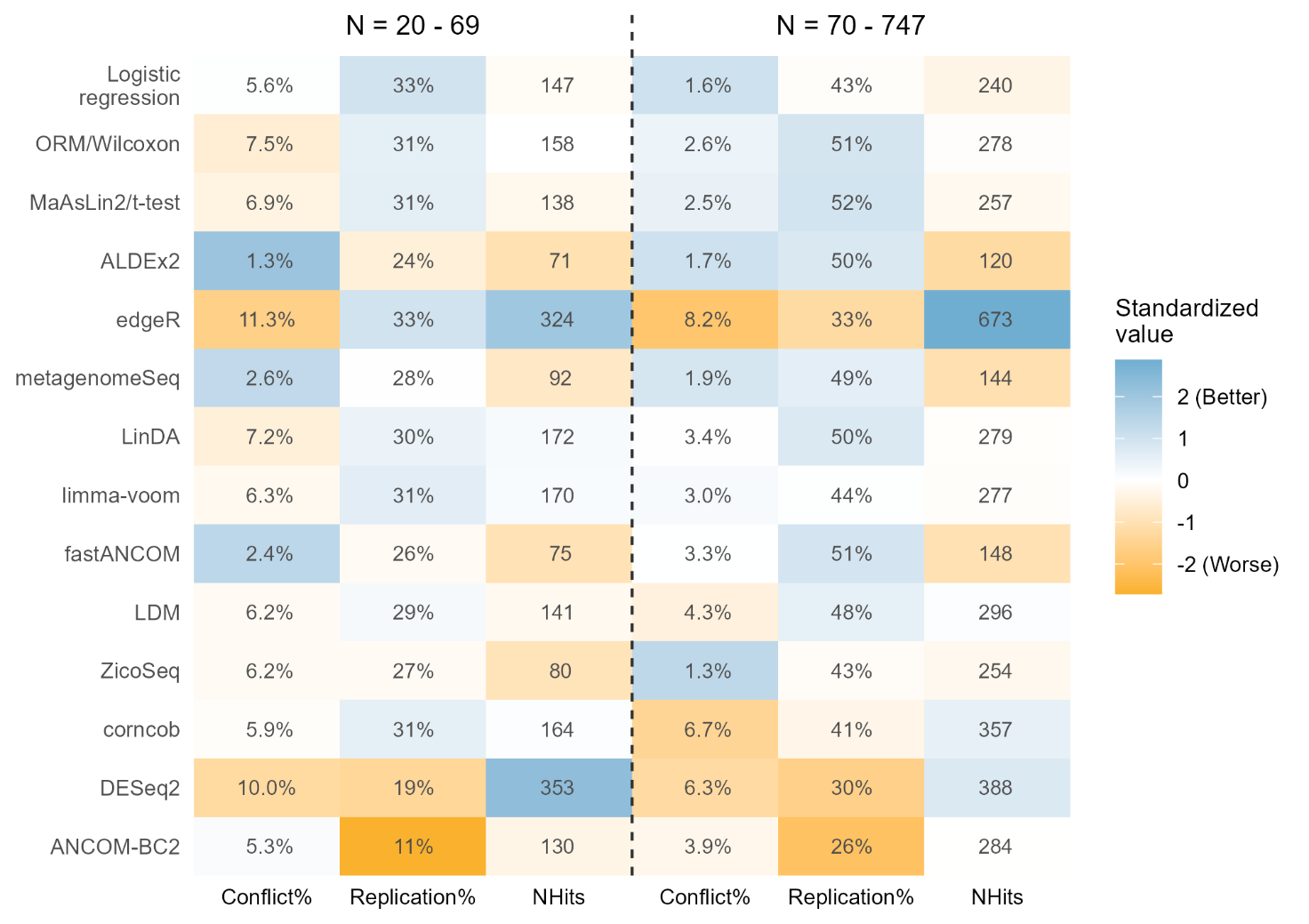


**Figure A17.2** The main results of the separate study analyses, stratified by sample size (N). The sample size refers to the number of subjects in the *exploratory* datasets. The larger datasets (N > 70) were used as the validation datasets also for the smaller datasets (20 <= N < 70). There were 17 and 20 exploratory datasets with N = 20 – 69 and N = 70 - 747, respectively. FDR level α = .05 was employed in the exploratory datasets.


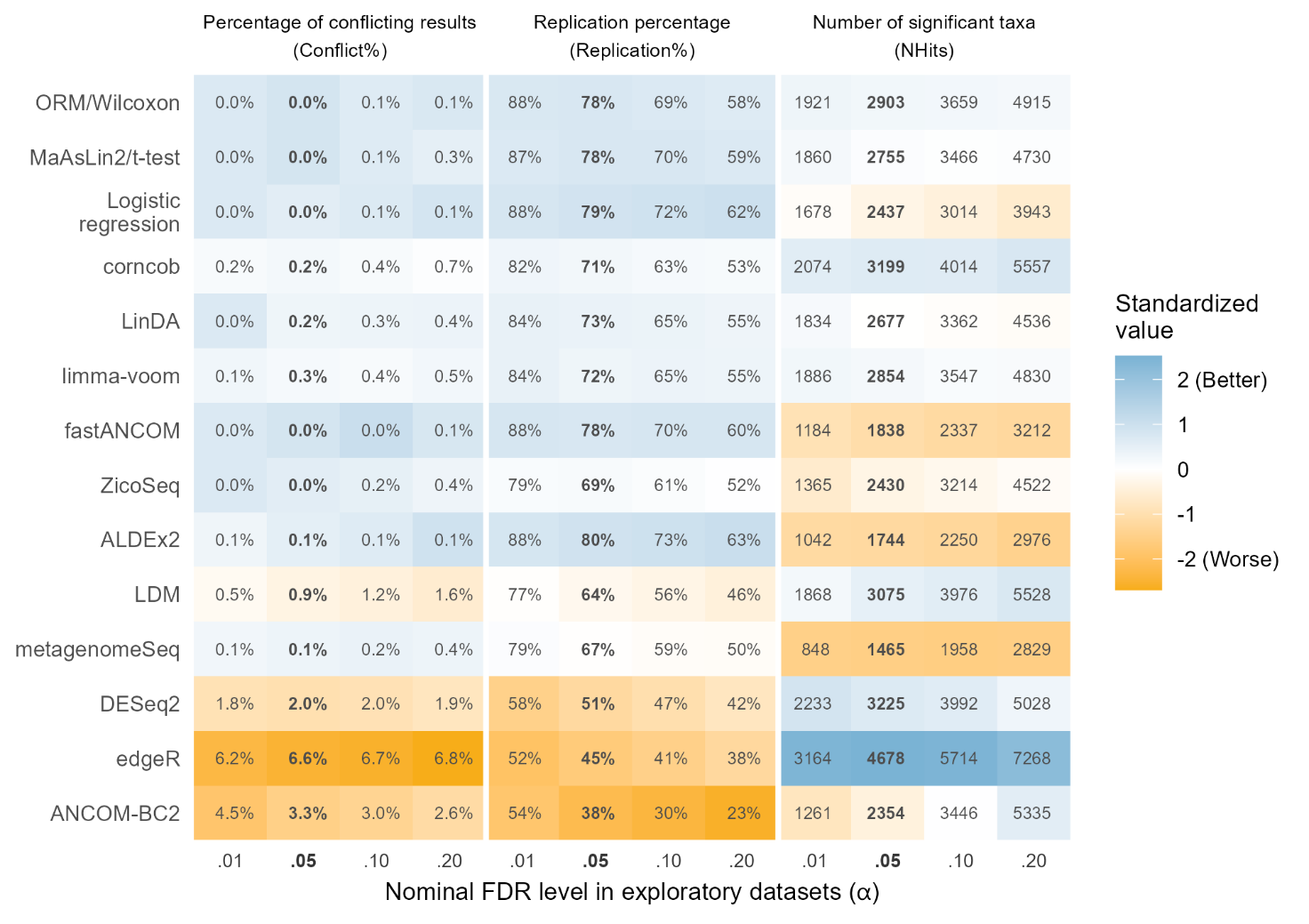


**Figure A18.1** The main results of the split-data analyses when exploratory and validation datasets with sample size N < 50 were filtered out (N referring to the number of subjects in a single exploratory or validation dataset). This corresponds to Figure 2 in the main text.


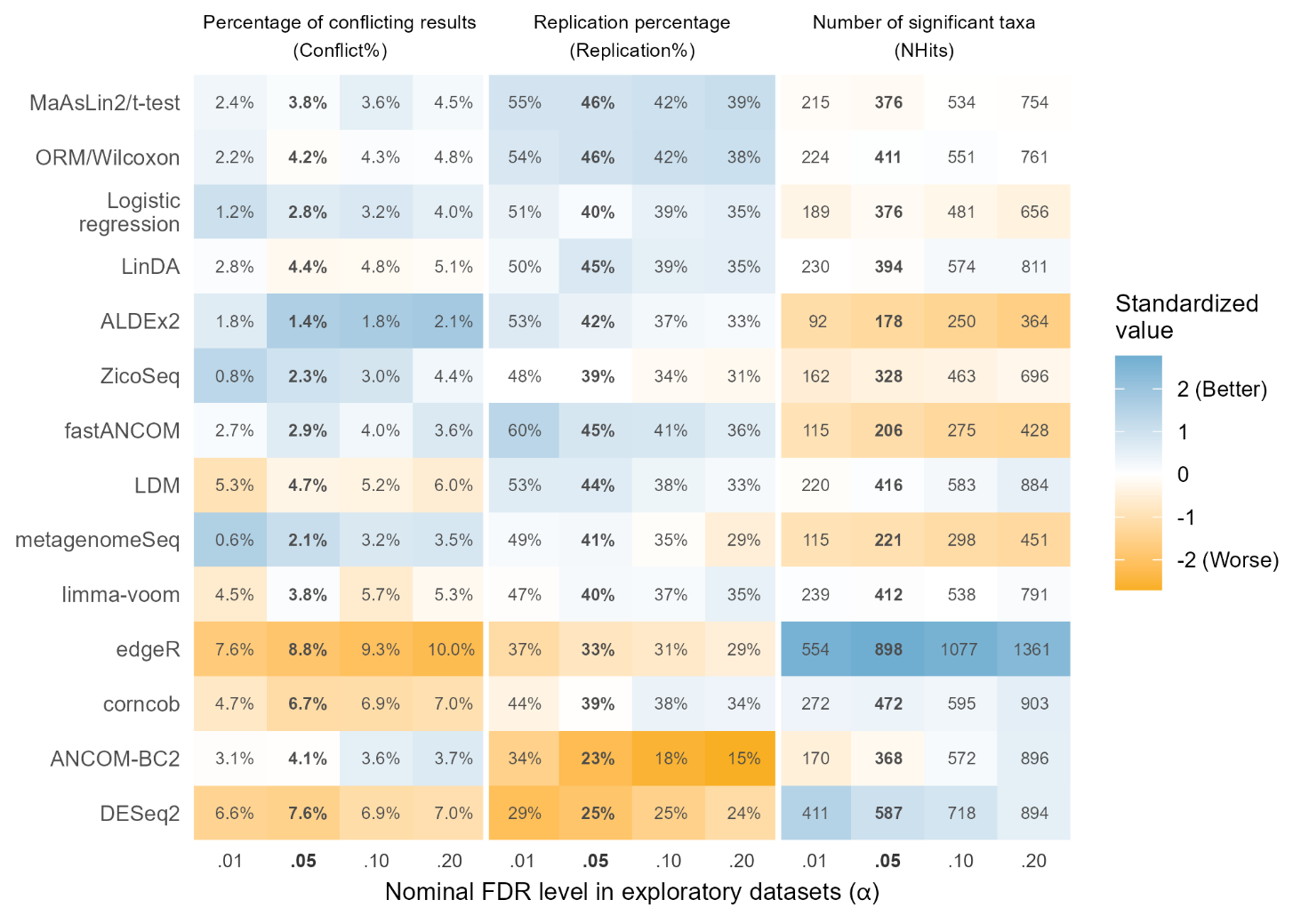


**Figure A18.2** The main results of the separate study analyses when exploratory and validation datasets with sample size N < 50 were filtered out. This corresponds to Figure 4 in the main text.


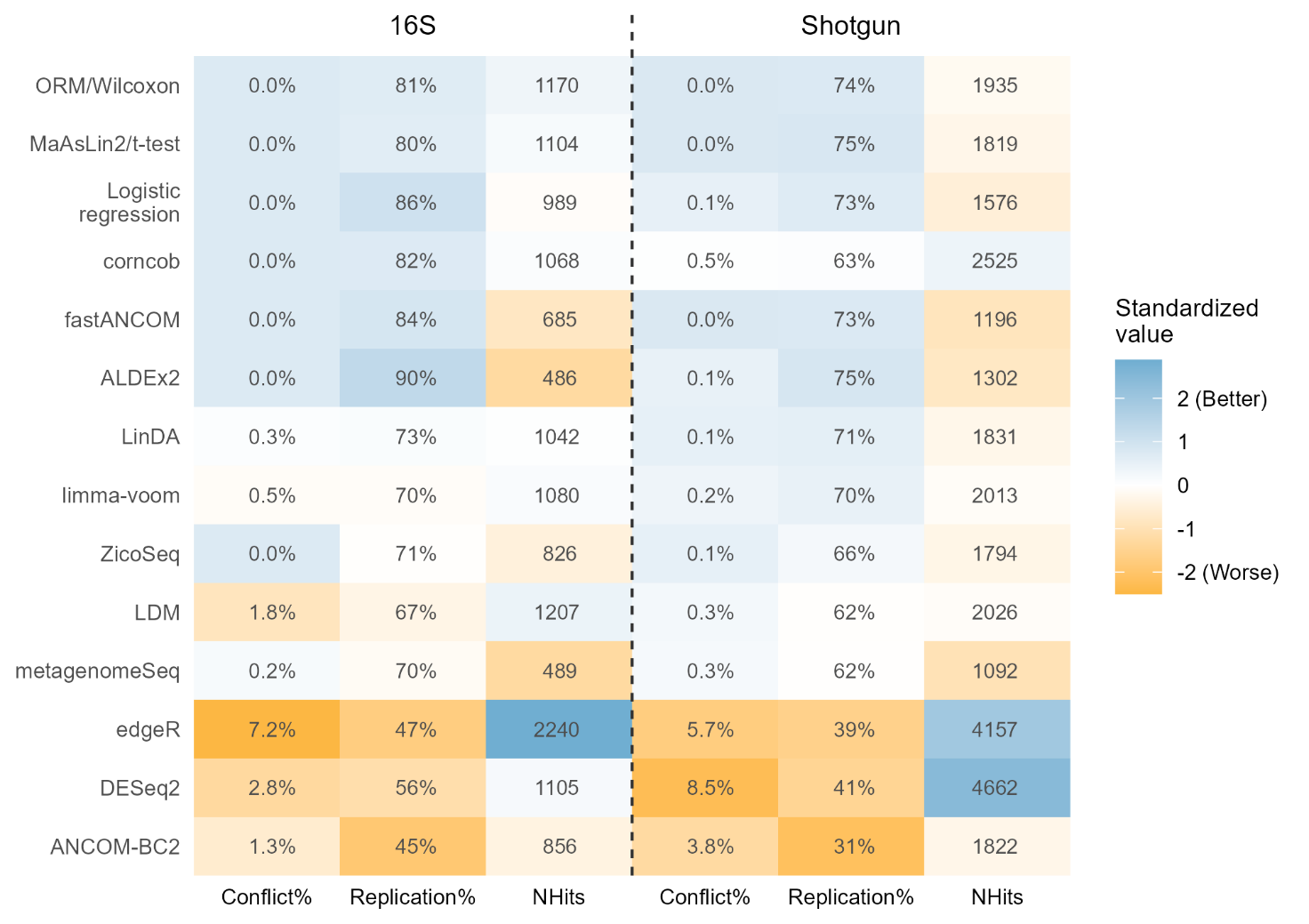


**Figure A19.1** The main results of the split-data analyses, stratified by sequencing type (16S or Shotgun). FDR level α = .05 was employed in the exploratory datasets.


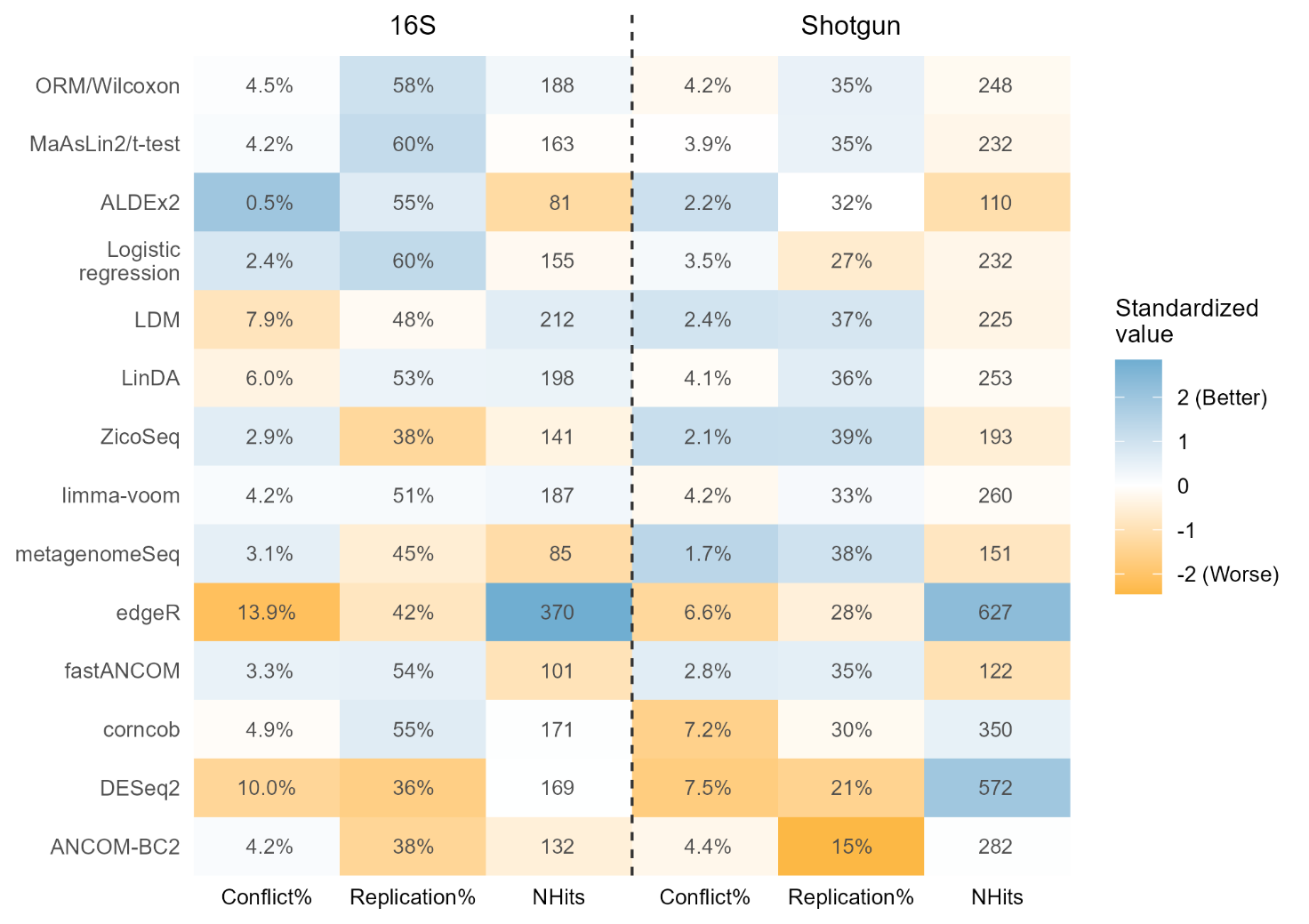


**Figure A19.2** The main results of the separate study analyses, stratified by sequencing type (16S or Shotgun). FDR level α = .05 was employed in the exploratory datasets.


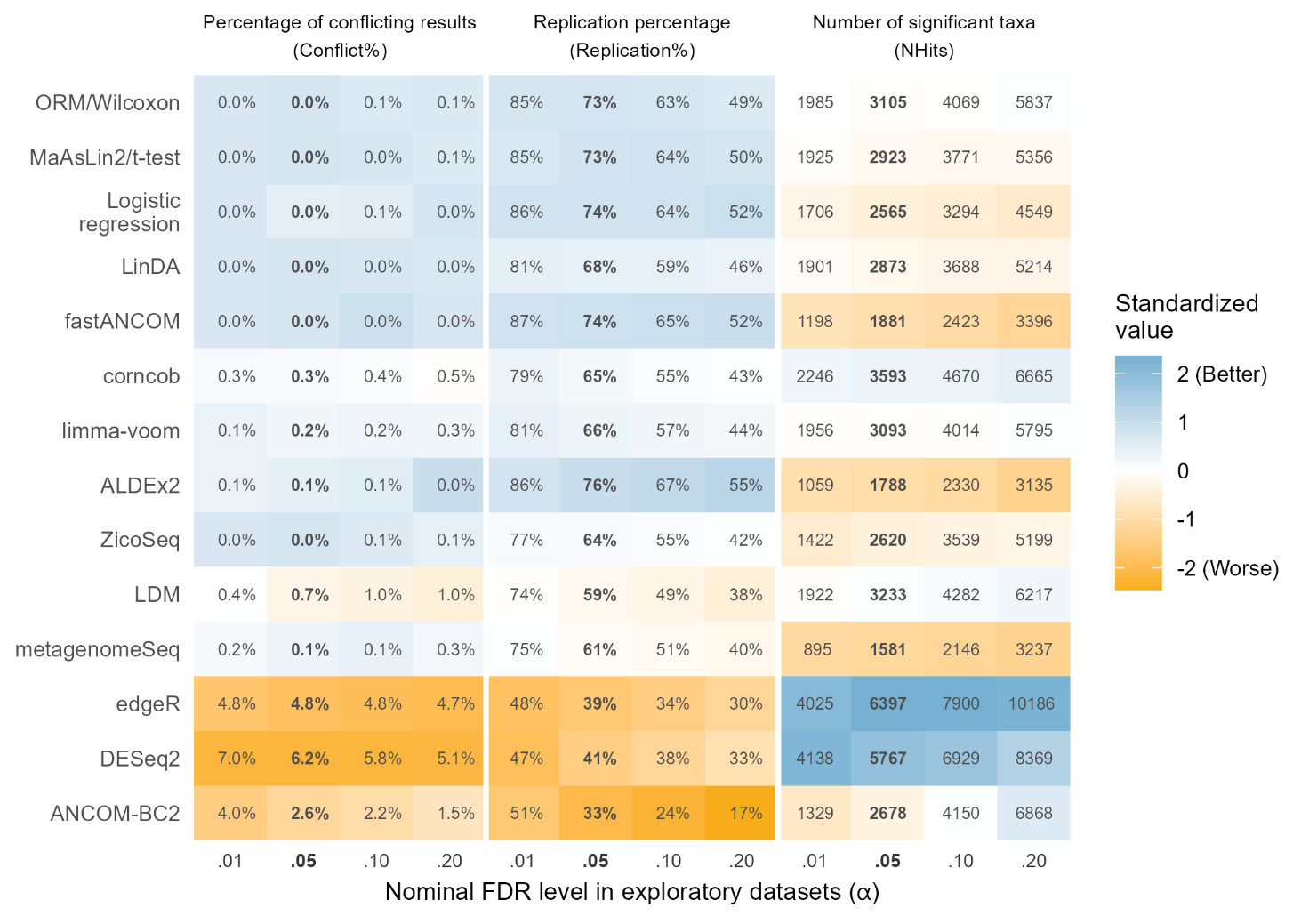


**Figure A20.1** The main results of the split-data analyses when p-values that were FDR adjusted over *candidate* taxa in the validation datasets were employed (instead of unadjusted p-values). That is, for each candidate taxon, the FDR adjusted p-value in the validation dataset was calculated by employing Benjamini-Hochberg method over the p-values of the candidate taxa. The taxon was then considered as statistically significant in the validation dataset if this FDR adjusted p-value was below .05.


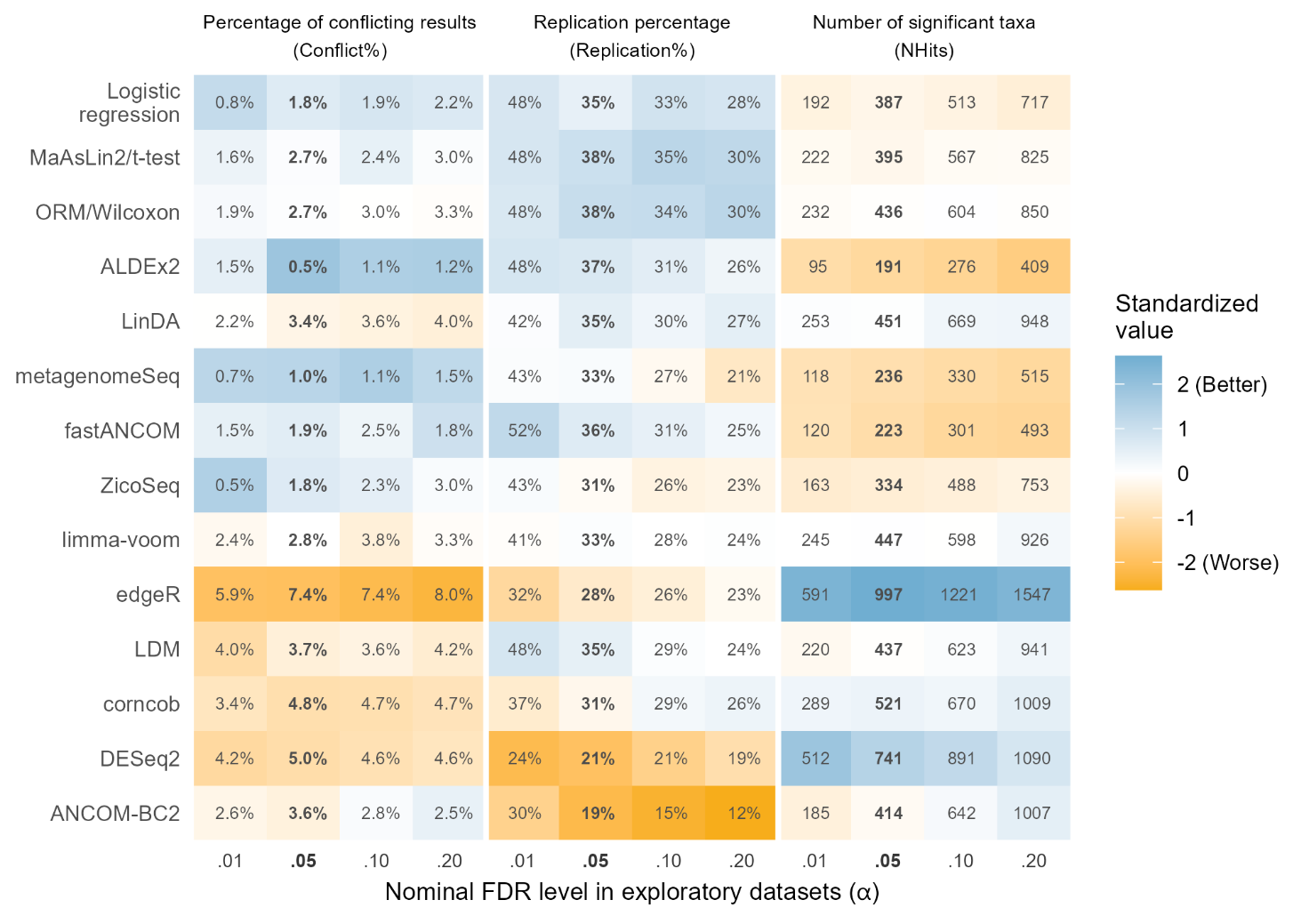


**Figure A20.2** The main results of the separate study analyses when p-values that were FDR adjusted over *candidate* taxa in the validation datasets were employed (instead of unadjusted p-values). That is, for each candidate taxon, the FDR adjusted p-value in the validation dataset was calculated by employing Benjamini-Hochberg method over the p-values of the candidate taxa. The taxon was then considered as statistically significant in the validation dataset if this FDR adjusted p-value was below .05.

**References**

[1] A. K. Alkanani *et al.*, ‘Alterations in Intestinal Microbiota Correlate With Susceptibility to Type 1 Diabetes’, *Diabetes*, vol. 64, no. 10, pp. 3510–3520, Oct. 2015, doi: 10.2337/db14-1847.

[2] N. T. Baxter, M. T. Ruffin, M. A. M. Rogers, and P. D. Schloss, ‘Microbiota-based model improves the sensitivity of fecal immunochemical test for detecting colonic lesions’, *Genome Med*, vol. 8, no. 1, p. 37, Dec. 2016, doi: 10.1186/s13073-016-0290-3.

[3] W. Chen, F. Liu, Z. Ling, X. Tong, and C. Xiang, ‘Human Intestinal Lumen and Mucosa-Associated Microbiota in Patients with Colorectal Cancer’, *PLoS One*, vol. 7, no. 6, p. e39743, Jun. 2012, doi: 10.1371/journal.pone.0039743.

[4] D. Gevers *et al.*, ‘The Treatment-Naive Microbiome in New-Onset Crohn’s Disease’, *Cell Host Microbe*, vol. 15, no. 3, pp. 382–392, Mar. 2014, doi: 10.1016/j.chom.2014.02.005.

[5] J. K. Goodrich *et al.*, ‘Human Genetics Shape the Gut Microbiome’, *Cell*, vol. 159, no. 4, pp. 789–799, Nov. 2014, doi: 10.1016/j.cell.2014.09.053.

[6] D.-W. Kang *et al.*, ‘Reduced Incidence of Prevotella and Other Fermenters in Intestinal Microflora of Autistic Children’, *PLoS One*, vol. 8, no. 7, p. e68322, Jul. 2013, doi: 10.1371/journal.pone.0068322.

[7] X. C. Morgan *et al.*, ‘Dysfunction of the intestinal microbiome in inflammatory bowel disease and treatment’, *Genome Biol*, vol. 13, no. 9, p. R79, 2012, doi: 10.1186/gb-2012-13-9-r79.

[8] M. Noguera-Julian *et al.*, ‘Gut Microbiota Linked to Sexual Preference and HIV Infection’, *EBioMedicine*, vol. 5, pp. 135–146, Mar. 2016, doi: 10.1016/j.ebiom.2016.01.032.

[9] E. Papa *et al.*, ‘Non-Invasive Mapping of the Gastrointestinal Microbiota Identifies Children with Inflammatory Bowel Disease’, *PLoS One*, vol. 7, no. 6, p. e39242, Jun. 2012, doi: 10.1371/journal.pone.0039242.

[10] F. Scheperjans *et al.*, ‘Gut microbiota are related to Parkinson’s disease and clinical phenotype’, *Movement Disorders*, vol. 30, no. 3, pp. 350–358, Mar. 2015, doi: 10.1002/mds.26069.

[11] J. U. Scher *et al.*, ‘Expansion of intestinal Prevotella copri correlates with enhanced susceptibility to arthritis’, *Elife*, vol. 2, Nov. 2013, doi: 10.7554/eLife.01202.

[12] A. M. Schubert *et al.*, ‘Microbiome Data Distinguish Patients with Clostridium difficile Infection and Non-C. difficile-Associated Diarrhea from Healthy Controls’, *mBio*, vol. 5, no. 3, Jul. 2014, doi: 10.1128/mBio.01021-14.

[13] P. Singh *et al.*, ‘Intestinal microbial communities associated with acute enteric infections and disease recovery’, *Microbiome*, vol. 3, no. 1, p. 45, Dec. 2015, doi: 10.1186/s40168-015-0109-2.

[14] J. S. Son *et al.*, ‘Comparison of Fecal Microbiota in Children with Autism Spectrum Disorders and Neurotypical Siblings in the Simons Simplex Collection’, *PLoS One*, vol. 10, no. 10, p. e0137725, Oct. 2015, doi: 10.1371/journal.pone.0137725.

[15] P. J. Turnbaugh *et al.*, ‘A core gut microbiome in obese and lean twins’, *Nature*, vol. 457, no. 7228, pp. 480–484, Jan. 2009, doi: 10.1038/nature07540.

[16] C. Vincent *et al.*, ‘Reductions in intestinal Clostridiales precede the development of nosocomial Clostridium difficile infection’, *Microbiome*, vol. 1, no. 1, p. 18, Dec. 2013, doi: 10.1186/2049-2618-1-18.

[17] T. Wang *et al.*, ‘Structural segregation of gut microbiota between colorectal cancer patients and healthy volunteers’, *ISME J*, vol. 6, no. 2, pp. 320–329, Feb. 2012, doi: 10.1038/ismej.2011.109.

[18] B. P. Willing *et al.*, ‘A Pyrosequencing Study in Twins Shows That Gastrointestinal Microbial Profiles Vary With Inflammatory Bowel Disease Phenotypes’, *Gastroenterology*, vol. 139, no. 6, pp. 1844-1854.e1, Dec. 2010, doi: 10.1053/j.gastro.2010.08.049.

[19] J. P. Zackular, M. A. M. Rogers, M. T. Ruffin, and P. D. Schloss, ‘The Human Gut Microbiome as a Screening Tool for Colorectal Cancer’, *Cancer Prevention Research*, vol. 7, no. 11, pp. 1112–1121, Nov. 2014, doi: 10.1158/1940-6207.CAPR-14-0129.

[20] G. Zeller *et al.*, ‘Potential of fecal microbiota for early-stage detection of colorectal cancer.’, *Mol Syst Biol*, vol. 10, no. 11, p. 766, Nov. 2014, doi: 10.15252/msb.20145645.

[21] Z. Zhang *et al.*, ‘Large-Scale Survey of Gut Microbiota Associated With MHE Via 16S rRNA-Based Pyrosequencing’, *American Journal of Gastroenterology*, vol. 108, no. 10, pp. 1601–1611, Oct. 2013, doi: 10.1038/ajg.2013.221.

[22] L. Zhu *et al.*, ‘Characterization of gut microbiomes in nonalcoholic steatohepatitis (NASH) patients: A connection between endogenous alcohol and NASH’, *Hepatology*, vol. 57, no. 2, pp. 601–609, Feb. 2013, doi: 10.1002/hep.26093.

[23] M. L. Zupancic *et al.*, ‘Analysis of the Gut Microbiota in the Old Order Amish and Its Relation to the Metabolic Syndrome’, *PLoS One*, vol. 7, no. 8, p. e43052, Aug. 2012, doi: 10.1371/journal.pone.0043052.

[24] J. R. Bedarf *et al.*, ‘Functional implications of microbial and viral gut metagenome changes in early stage L-DOPA-naïve Parkinson’s disease patients.’, *Genome Med*, vol. 9, no. 1, p. 39, Apr. 2017, doi: 10.1186/s13073-017-0428-y.

[25] Q. Feng *et al.*, ‘Gut microbiome development along the colorectal adenoma-carcinoma sequence.’, *Nat Commun*, vol. 6, p. 6528, Mar. 2015, doi: 10.1038/ncomms7528.

[26] A. Gupta *et al.*, ‘Association of Flavonifractor plautii, a Flavonoid-Degrading Bacterium, with the Gut Microbiome of Colorectal Cancer Patients in India’, *mSystems*, vol. 4, no. 6, Dec. 2019, doi: 10.1128/MSYSTEMS.00438-19.

[27] A. B. Hall *et al.*, ‘A novel Ruminococcus gnavus clade enriched in inflammatory bowel disease patients.’, *Genome Med*, vol. 9, no. 1, p. 103, Nov. 2017, doi: 10.1186/s13073-017-0490-5.

[28] G. D. Hannigan, M. B. Duhaime, M. T. Ruffin, C. C. Koumpouras, and P. D. Schloss, ‘Diagnostic Potential and Interactive Dynamics of the Colorectal Cancer Virome.’, *mBio*, vol. 9, no. 6, Nov. 2018, doi: 10.1128/mBio.02248-18.

[29] A. Heintz-Buschart *et al.*, ‘Integrated multi-omics of the human gut microbiome in a case study of familial type 1 diabetes.’, *Nat Microbiol*, vol. 2, p. 16180, Oct. 2016, doi: 10.1038/nmicrobiol.2016.180.

[30] U. Z. Ijaz *et al.*, ‘The distinct features of microbial “dysbiosis” of Crohn’s disease do not occur to the same extent in their unaffected, genetically-linked kindred.’, *PLoS One*, vol. 12, no. 2, p. e0172605, 2017, doi: 10.1371/journal.pone.0172605.

[31] Z. Jie *et al.*, ‘The gut microbiome in atherosclerotic cardiovascular disease.’, *Nat Commun*, vol. 8, no. 1, p. 845, Oct. 2017, doi: 10.1038/s41467-017-00900-1.

[32] F. H. Karlsson *et al.*, ‘Gut metagenome in European women with normal, impaired and diabetic glucose control.’, *Nature*, vol. 498, no. 7452, pp. 99–103, Jun. 2013, doi: 10.1038/nature12198.

[33] J. Li *et al.*, ‘An integrated catalog of reference genes in the human gut microbiome.’, *Nat Biotechnol*, vol. 32, no. 8, pp. 834–41, Aug. 2014, doi: 10.1038/nbt.2942.

[34] J. Li *et al.*, ‘Gut microbiota dysbiosis contributes to the development of hypertension.’, *Microbiome*, vol. 5, no. 1, p. 14, Feb. 2017, doi: 10.1186/s40168-016-0222-x.

[35] D. Nagy-Szakal *et al.*, ‘Fecal metagenomic profiles in subgroups of patients with myalgic encephalomyelitis/chronic fatigue syndrome.’, *Microbiome*, vol. 5, no. 1, p. 44, Apr. 2017, doi: 10.1186/s40168-017-0261-y.

[36] H. B. Nielsen *et al.*, ‘Identification and assembly of genomes and genetic elements in complex metagenomic samples without using reference genomes.’, *Nat Biotechnol*, vol. 32, no. 8, pp. 822–8, Aug. 2014, doi: 10.1038/nbt.2939.

[37] J. Qin *et al.*, ‘A metagenome-wide association study of gut microbiota in type 2 diabetes.’, *Nature*, vol. 490, no. 7418, pp. 55–60, Oct. 2012, doi: 10.1038/nature11450.

[38] N. Qin *et al.*, ‘Alterations of the human gut microbiome in liver cirrhosis.’, *Nature*, vol. 513, no. 7516, pp. 59–64, Sep. 2014, doi: 10.1038/nature13568.

[39] F. Raymond *et al.*, ‘The initial state of the human gut microbiome determines its reshaping by antibiotics.’, *ISME J*, vol. 10, no. 3, pp. 707–20, Mar. 2016, doi: 10.1038/ismej.2015.148.

[40] M. A. Rubel *et al.*, ‘Lifestyle and the presence of helminths is associated with gut microbiome composition in Cameroonians.’, *Genome Biol*, vol. 21, no. 1, p. 122, May 2020, doi: 10.1186/s13059-020-02020-4.

[41] K. Sankaranarayanan *et al.*, ‘Gut Microbiome Diversity among Cheyenne and Arapaho Individuals from Western Oklahoma.’, *Curr Biol*, vol. 25, no. 24, pp. 3161–9, Dec. 2015, doi: 10.1016/j.cub.2015.10.060.

[42] M. Schirmer *et al.*, ‘Dynamics of metatranscription in the inflammatory bowel disease gut microbiome.’, *Nat Microbiol*, vol. 3, no. 3, pp. 337–346, Mar. 2018, doi: 10.1038/s41564-017-0089-z.

[43] A. M. Thomas *et al.*, ‘Metagenomic analysis of colorectal cancer datasets identifies cross-cohort microbial diagnostic signatures and a link with choline degradation.’, *Nat Med*, vol. 25, no. 4, pp. 667–678, Apr. 2019, doi: 10.1038/s41591-019-0405-7.

[44] E. Vogtmann *et al.*, ‘Colorectal Cancer and the Human Gut Microbiome: Reproducibility with Whole-Genome Shotgun Sequencing.’, *PLoS One*, vol. 11, no. 5, p. e0155362, 2016, doi: 10.1371/journal.pone.0155362.

[45] J. Wirbel *et al.*, ‘Meta-analysis of fecal metagenomes reveals global microbial signatures that are specific for colorectal cancer.’, *Nat Med*, vol. 25, no. 4, pp. 679–689, Apr. 2019, doi: 10.1038/s41591-019-0406-6.

[46] H. Xie *et al.*, ‘Shotgun Metagenomics of 250 Adult Twins Reveals Genetic and Environmental Impacts on the Gut Microbiome.’, *Cell Syst*, vol. 3, no. 6, pp. 572-584.e3, Dec. 2016, doi: 10.1016/j.cels.2016.10.004.

[47] S. Yachida *et al.*, ‘Metagenomic and metabolomic analyses reveal distinct stage-specific phenotypes of the gut microbiota in colorectal cancer.’, *Nat Med*, vol. 25, no. 6, pp. 968–976, Jun. 2019, doi: 10.1038/s41591-019-0458-7.

[48] Z. Ye *et al.*, ‘A metagenomic study of the gut microbiome in Behcet’s disease.’, *Microbiome*, vol. 6, no. 1, p. 135, Aug. 2018, doi: 10.1186/s40168-018-0520-6.

[49] J. Yu *et al.*, ‘Metagenomic analysis of faecal microbiome as a tool towards targeted non-invasive biomarkers for colorectal cancer.’, *Gut*, vol. 66, no. 1, pp. 70–78, Jan. 2017, doi: 10.1136/gutjnl-2015-309800.

[50] F. Zhu *et al.*, ‘Metagenome-wide association of gut microbiome features for schizophrenia.’, *Nat Commun*, vol. 11, no. 1, p. 1612, Mar. 2020, doi: 10.1038/s41467-020-15457-9.

[51] M. P. Nixon, G. B. Gloor, and J. D. Silverman, ‘Beyond Normalization: Incorporating Scale Uncertainty in Microbiome and Gene Expression Analysis’, *bioRxiv*, p. 2024.04.01.587602, Apr. 2024, doi: 10.1101/2024.04.01.587602.

[52] Y.-J. Hu and G. A. Satten, ‘Compositional analysis of microbiome data using the linear decomposition model (LDM)’, *bioRxiv*, p. 2023.05.26.542540, May 2023, doi: 10.1101/2023.05.26.542540.

[53] D. Firth, ‘Bias Reduction of Maximum Likelihood Estimates’, *Biometrika*, vol. 80, no. 1, p. 27, Mar. 1993, doi: 10.2307/2336755.

[54] J. T. Nearing *et al.*, ‘Microbiome differential abundance methods produce different results across 38 datasets’, *Nat Commun*, vol. 13, no. 1, p. 342, Jan. 2022, doi: 10.1038/s41467-022-28034-z.

[55] D. S. Clausen and A. D. Willis, ‘Estimating Fold Changes from Partially Observed Outcomes with Applications in Microbial Metagenomics’, *ArXiv*, Feb. 2024, Accessed: Mar. 05, 2025. [Online]. Available: https://arxiv.org/abs/2402.05231v1

[56] B. Brill, A. Amir, and R. Heller, ‘Testing for differential abundance in compositional counts data, with application to microbiome studies’, *Ann Appl Stat*, vol. 16, no. 4, pp. 2648–2671, 2022.

[57] Y. Hu, G. A. Satten, and Y. J. Hu, ‘LOCOM: A logistic regression model for testing differential abundance in compositional microbiome data with false discovery rate control’, *Proc Natl Acad Sci U S A*, vol. 119, no. 30, p. e2122788119, Jul. 2022, doi: 10.1073/PNAS.2122788119/SUPPL_FILE/PNAS.2122788119.SAPP.PDF.

[58] J. T. Barlow, S. R. Bogatyrev, and R. F. Ismagilov, ‘A quantitative sequencing framework for absolute abundance measurements of mucosal and lumenal microbial communities’, *Nature Communications 2020 11:1*, vol. 11, no. 1, pp. 1–13, May 2020, doi: 10.1038/s41467-020-16224-6.
